# The effective rate of influenza reassortment is limited during human infection

**DOI:** 10.1371/journal.ppat.1006203

**Published:** 2017-02-07

**Authors:** Ashley Sobel Leonard, Micah T. McClain, Gavin J. D. Smith, David E. Wentworth, Rebecca A. Halpin, Xudong Lin, Amy Ransier, Timothy B. Stockwell, Suman R. Das, Anthony S. Gilbert, Rob Lambkin-Williams, Geoffrey S. Ginsburg, Christopher W. Woods, Katia Koelle, Christopher J. R. Illingworth

**Affiliations:** 1 Department of Biology, Duke University, Durham, North Carolina, United States of America; 2 Duke Center for Applied Genomics and Precision Medicine, Durham, North Carolina, United States of America; 3 Programme in Emerging Infectious Diseases, Duke-NUS Medical School, Singapore; 4 J. Craig Venter Institute, Rockville, Maryland, United States of America; 5 hVivo PLC, The QMB Innovation Centre, Queen Mary, University of London, London, United Kingdom; 6 Department of Genetics, University of Cambridge, Cambridge, United Kingdom; 7 Department of Applied Maths and Theoretical Physics, Centre for Mathematical Sciences, Wilberforce Road, University of Cambridge, Cambridge, United Kingdom; University of Michigan, UNITED STATES

## Abstract

We characterise the evolutionary dynamics of influenza infection described by viral sequence data collected from two challenge studies conducted in human hosts. Viral sequence data were collected at regular intervals from infected hosts. Changes in the sequence data observed across time show that the within-host evolution of the virus was driven by the reversion of variants acquired during previous passaging of the virus. Treatment of some patients with oseltamivir on the first day of infection did not lead to the emergence of drug resistance variants in patients. Using an evolutionary model, we inferred the effective rate of reassortment between viral segments, measuring the extent to which randomly chosen viruses within the host exchange genetic material. We find strong evidence that the rate of effective reassortment is low, such that genetic associations between polymorphic loci in different segments are preserved during the course of an infection in a manner not compatible with epistasis. Combining our evidence with that of previous studies we suggest that spatial heterogeneity in the viral population may reduce the extent to which reassortment is observed. Our results do not contradict previous findings of high rates of viral reassortment *in vitro* and in small animal studies, but indicate that in human hosts the effective rate of reassortment may be substantially more limited.

## Author summary

The influenza virus is an important cause of disease in the human population. During the course of an infection the virus can evolve rapidly. An important mechanism of viral evolution is reassortment, whereby different segments of the influenza genome are shuffled with other segments, producing new viral combinations. Here we study natural selection and reassortment during the course of infections occurring in human hosts. Examining viral genome sequence data from these infections, we note that genetic variants that were acquired during the growth of viruses in culture are selected against in the human host. In addition, we find evidence that the effective rate of reassortment is low. We suggest that the spatial separation between viruses in different parts of the host airway may limit the extent to which genetically distinct segments reassort with one another. Within the global population of influenza viruses, reassortment remains an important factor. However, reassortment is not so rapid as to exclude the possibility of interactions between genome segments affecting the course of influenza evolution during a single infection.

## Introduction

Genome sequencing has provided multiple insights into the evolution of the influenza virus. At the global level, sequences collected from circulating strains of the virus have enabled the identification of codons in the virus evolving under positive selection [1, 2], and demonstrated the importance of interactions between selected alleles [3–5]. Collected sequence data have been used to understand the global migration dynamics of different seasonal strains [6–8] and to make predictions for the future evolution of the virus [9–11]. Considering infection at the level of a single host, sequencing technology has been used to explore the evolution of the influenza virus over the course of a single infection and during the process of viral transmission [12, 13]. Studies of this form have highlighted both the potential for rapid changes in sequence composition within a series of transmission events, as well as diversity in the antigenic properties of a viral population within a single host [14–16]. Experiments conducted in ferrets have considered the within-host evolution and transmission of novel viral strains [17–20].

While some phenotypic properties of the influenza virus can be understood in terms of genetic changes occurring within a single gene [21], interactions between genes have increasingly been identified as of importance for viral evolution. For example, evidence for epistasis in influenza has been found between variants both within single influenza genes [22–24] and across different gene segments [25–27], including the occurrence of permissive mutations which facilitate protein evolution and immune escape. A key multi-gene effect in influenza dynamics is the process of reassortment, whereby distinct influenza viruses within a single cell produce viruses with novel combinations of genome segments, potentially leading to the emergence of new, beneficial strains [28]; evidence for frequent reassortment has been identified in global viral populations [29]. While precise rates of reassortment are hard to quantify, both *in vitro* and *in vivo* experiments have suggested that reassortment between compatible strains of influenza occurs readily [30, 31]. Although the extent of reassortment in experimentally induced infections can depend upon the number of viruses received by a host [32], viral transmission gives a sufficient dose for within-host reassortment to be observed in small animal infection [33], suggesting that reassortment plays an important role in within-host viral dynamics.

Quantitative modelling of within-host influenza infection has a long history [34], but it is only recently that sequencing studies have provided the necessary information to fit evolutionary models to data. Standard techniques for detecting selection, such as dN/dS, are not appropriate for such populations, in which the time-scale for evolution is extremely short [35, 36]. Homologous recombination is rare [37–39], or potentially non-existent, such that selection acting upon one variant may, via linkage disequilibrium, cause changes in allele frequency across a single segment. Under the influence of selection, a population evolves according to a ‘fitness landscape’, which describes the expected within-host growth rate of each virus as a function of viral genotype [40]. Given time-resolved sequence data, approaches which account for the haplotype structure of the viral population have been applied in order to infer the core components of this landscape as it influences viral evolution [41, 42]. However, even in these inferences, the potential role of reassortment has not been considered. Given anything other than a rapid rate of reassortment, linkage disequilibrium between alleles in different segments may have an effect on viral dynamics.

Studies of within-host influenza growth and transmission have been conducted in a variety of animals, including birds, dogs, ferrets, pigs, horses and small mammals [13, 43–45]. Such studies are of great inherent importance, and have long been acknowledged as contributing to our understanding of human infections [46]. However, from the specific perspective of human health, while ferrets in particular provide a valuable model for understanding influenza infection [47, 48], studies conducted in human hosts remain the ultimate reference point.

We here describe results from one of the first studies to examine influenza evolution in humans from the perspective of time-resolved viral sequence data, which describe in detail the process of viral evolution as it occurs. Using longitudinal viral sequence data collected from subjects in two related influenza challenge studies (described in previous publications [15, 49–54]) we evaluate how fitness effects shape within-host viral evolution. Using a maximum likelihood approach, we infer the location and magnitude of the selective forces underlying observed changes in the genotypic structure of the viral population. Capturing reassortment in real time on the scale of an infected individual has been noted as a difficult task [55]. Here, using an inference method which incorporates the effect of linkage disequilibrium between alleles on distinct viral segments, we estimate the effective rate of reassortment within the viral population in human subjects on the basis of changes in the genetic composition of the viral population observed over time.

On the basis of the collected data, we infer that selection acting upon variants in the HA, NP, and PA viral genes is responsible for driving within-host evolution in the subjects from which data were collected. Further, we find strong evidence to suggest that the effective rate of reassortment between variants found on different gene segments is limited, such that linkage disequilibrium between alleles on different segments is maintained throughout the course of an infection. The inferences derived via our model are consistent with previous findings that the extent of within-host recombination is linked to the dose of virus received by a patient. Our results are reconcilable with reports of high reassortment rates in *in vitro* and small animal studies given a scenario in which the absolute rate of reassortment is high, but the within-host influenza population is spatially distributed, forming a metapopulation within a patient. We conclude that interactions between variants on different viral segments may substantially affect within-host viral evolution.

## Results

### Model

**Fig 1:**
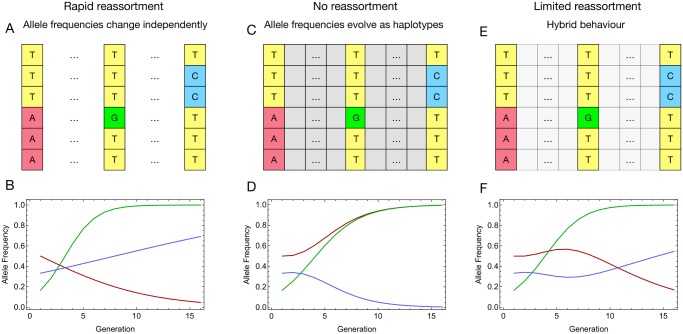
Patterns of allele frequency change are altered by the presence or absence of reassortment. **A.** Representation of a three-locus system with large population size, and in which rapid reassortment wipes out linkage disequilibrium between alleles at different loci. The ‘A’ variant (red) is under mild negative selection, the ‘G’ variant (green) is under strong positive selection, while the ‘C’ variant is under weak positive selection. **B.** Given the rapid rate of reassortment, alleles at each locus evolve deterministically over time in accordance with the selection acting upon them. **C.** Representation of the same system, now with no reassortment. Alleles now evolve according the changes in the frequencies of full sequences in the population; each horizontal row indicates a set of sequences with one of four distinct three-locus genotypes. **D.** While the initial state of the system, and magnitudes of selection are unchanged, the fates of the red and blue variants are reversed. The AGT genotype is the fittest of the four genotypes; in a process known as genetic hitchhiking, the deleterious red variant is carried by the green to fixation. In a process known as clonal interference, the beneficial blue variant is wiped out in the competition. **E.** Representation of the same system in which there is finite, but limited reassortment between the variants. **F.** Given limited reassortment, the association between the red and green variants is gradually eroded so that the red variant is lost over time. After an initial decline, formation of the maximally fit TGC haplotype leads to the blue variant increasing in frequency over time.

Our approach to estimating reassortment rates is based upon some elementary principles of population genetics. Given the relatively short length of an influenza infection, and the large number of viruses created during that infection, changes in the viral population are driven by the influence of selection. Selection changes allele frequencies over time, favouring individuals with variants that grant beneficial traits to the virus. However, the manner in which allele frequencies changes under selection depends upon whether variants at different nucleotides are free to change in a manner independent from one another, or whether they are linked together, perhaps by physical linkage, through epistatic effects, or via a lack of reassortment. Examples of how different reassortment rates affect the evolution of a system are shown in Fig 1. Here we exploit this idea to make the converse step, using observed changes in the genetic composition of multiple viral populations to infer the rate of reassortment within human subjects. Our model exploits the fact that, within a single segment of influenza, short sequence reads may potentially describe alleles at more than one locus. Such reads grant direct information into the associations between alleles in a single segment. These multi-locus data can be exploited to gain a clearer view of the evolution of the population; [42].

Under our approach, we first identify sites in the influenza genome at which polymorphism exists, and at which the minor allele frequency changes significantly over time. The set of observed polymorphisms is used to construct a set of potential virus-wide haplotypes, from which variant alleles are observed. We then construct a model under which the frequencies of viral haplotypes change in frequency over time under mutation and selection, and under the effect of reassortment between viral segments. A likelihood optimisation method is used to fit the model to the data under a wide range of possible rates of reassortment, allowing an inference to be made of the effective rate of reassortment between segments. Full details of our model are given in the Methods section.

### Data summary

The data used for this study were next-generation sequencing samples generated from two human challenge studies with influenza A/Wisconsin/67/2005(H3N2) virus [56]. Briefly, individuals enrolled in these studies were inoculated intranasally with an influenza H3N2 viral inoculum and monitored throughout maximal symptom development, a total of 7 days. The two studies differed in the treatment given to individuals. In the first study, individuals received standard treatment, with oseltamivir being administered on the evening of the 5th day post-challenge. In the second study, individuals received either standard treatment or early treatment, defined as the administration of oseltamivir on the evening of the 1st day post-challenge. As such, subjects were divided into two cohorts, a ‘standard treatment’ cohort, and an ‘early treatment’ cohort (Table 1). The viral inoculum used for both studies consisted of a genetically heterogeneous population of virus generated from passaged viral reference strain A/Wisconsin/67/2005(H3N2). The virus was passaged 3X in chicken cells, 4X in eggs and 2X in vero cells. Subjects were challenged with doses of the viral inoculum ranging from 3.08–6.41 log_10_(TCID_50_/ml) [57]. Previous analysis of this experiment showed there was no association between the inoculum dose and whether the challenge subject became infected [58]. For those subjects who were infected, there was no association between the inoculum dose and the degree of disease symptoms or the magnitude/duration of viral shedding [58].

**Table 1:** Challenge study subjects.

Subject	Experiment	Cohort	Inoculum (log_10_TCID_50_)ml^−1^	Symptomatic	Seroconversion
Flu001	1	Standard	6.41	Y	Y
Flu006	1	Standard	5.25	Y	Y
Flu012	1	Standard	4.41	Y	Y
Flu013	1	Standard	3.8	N	Y
Flu5001	2	Early	5.5	Y	Y
Flu5002	2	Early	5.5	Y	Y
Flu5004	2	Standard	5.5	Y	Y
Flu5006	2	Early	5.5	Y	N
Flu5007	2	Early	5.5	N	Y
Flu5018	2	Early	5.5	Y	Y
Flu5019	2	Early	5.5	Y	Y
Flu5020	2	Standard	5.5	Y	Y
Flu5021	2	Standard	5.5	N	Y

Nasal wash samples were collected daily and tested for the presence of virus using culture or quantitative PCR. Those samples containing virus (a total of 60 samples out of 266 samples obtained) and the viral inoculum, were sequenced at the JCVI, as described in [59, 60]. The next generation sequencing pipeline at the JCVI uses both the Illumina HiSeq 2000 and Ion Torrent platforms to compensate for platform specific errors [19]. The sequences from both platforms were used in this analysis. Sequences were obtained for 42 of the nasal wash samples from 17 subjects. As this analysis infers selection based upon on the trajectory of variant frequencies over the course of infection, we excluded samples from 4 individuals (Flu008, Flu010, Flu015 and Flu5017) who were only sequenced at one time-point. The remaining dataset consisted of 38 samples from 13 individuals (six of whom received early treatment and seven of whom received standard treatment), with between two and five samples per subject. Data showing the distribution of read lengths and the depth of sequencing are shown in S1 and S2 Figs. Sequence data are available from the SRA with the project accession number SRP091397.

### Single locus model

**Fig 2:**
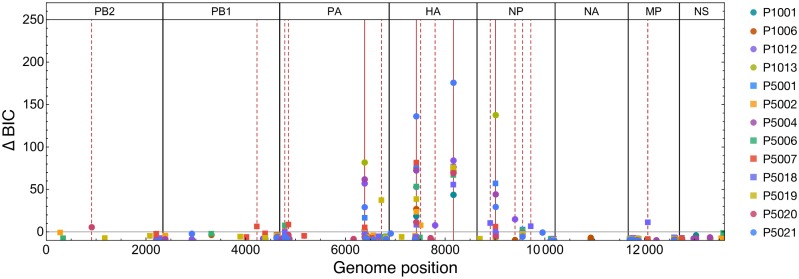
Single-locus scan for alleles potentially under selection. Bayesian Information Criterion (BIC) differences between the best selected model and the neutral model at each locus in the genome. Differences are reported for loci at which polymorphism was detected. A positive BIC difference indicates a weight of evidence in favour of selection, calculated using a single-locus model, applied to data from a single individual. Loci with a positive BIC difference are highlighted with vertical red dotted lines. Solid red vertical lines show loci at which selection was later identified using a multi-locus model with data from all individuals. Circles denote results from individuals receiving standard treatment; squares denote results from individuals receiving early treatment.

Considering data from all time-points and all individuals, polymorphisms were identified at a total of 110 loci (nucleotide sites) across the influenza genome. Allele frequencies at these loci measured across time, which we refer to as trajectories, are shown in S3–S10 Figs. The inoculum sample was used as a proxy measurement of the population establishing infection within each host. Trajectories were assessed using a single-locus model of allele frequency change (c.f. [61]), looking for statistically significant deviation from a neutral model, under which the frequency of an allele remained constant over time. Applying this model a total of 16 loci, distributed across six of the eight influenza genome segments, showed potential evidence of non-neutral allele frequency change (Fig 2; equivalent data by segment are shown in S11 Fig). The absence of evidence for significant allele frequency change in either the NA or NS segments implies the absence of strong positive selection in each case. In NA the emergence of drug resistance mutations, notably the amino acid substitution H274T arising from the nucleotide variant C820T, has previously been reported following prolonged oseltamivir treatment in an immunocompromised host [62]. However, no such event was observed here, in either the standard or the early-treatment populations.

### Single-gene, multi-locus model

A single-segment, multi-locus model (SGML) [42] was applied to alleles at the potentially selected loci, accounting for the correlations between alleles that arise due to the lack of homologous recombination in influenza segments. Under this method, short-read data from each segment are used to identify potential haplotypes spanning the segment [63]. Considering potential haplotypes, we note that in a case where two alleles are observed at each of *n* different loci, a total of 2^*n*^ potential haplotypes may exist. However, where reads span multiple loci, and these multi-locus reads indicate limited diversity across sites, the number of potential haplotypes required by the model may be reduced. Potential segment-wide haplotypes were used under a model of mutation and selection to construct the most parsimonious model of selection acting within each segment, inferring the initial frequency of each haplotype and noting that selection at one locus may explain a change in allele frequency at another. Under the assumption of a consistent model of selection acting across all individuals, we identified potential evidence for selection at 9 loci, found in the HA, NP, PA, and PB2 segments (Table 2). A negative epistatic interaction between two variants in the HA segment was also inferred; negative epistasis implies that each variant incurs a fitness penalty in the presence of the other, in addition to its inherent effect upon viral fitness. This reduction in the number of potentially selected sites confirms the importance of linkage disequilibrium in influenza dynamics; changes in allele frequencies that under the single locus model appeared to result from the direct effect of selection were often better explained by the indirect effect of selection acting at another locus. For example, four different loci in the PA segment were identified as having significant changes in allele frequency using the single locus model, but when the segment was considered as a whole, selection at locus 1680 was sufficient to explain the changes observed across all four loci; adding selection at a second locus did not significantly improve the ability of the multi-locus model to fit the data.

**Table 2:** Inferred fitness coefficients for the single-segment, multi-locus model across all individuals. Best fitting models are shown for each number of parameters; the best fitting model for data from each segment is highlighted in bold text. The parameter χ denotes an epistatic interaction between alleles.

Model	Likelihood	BIC	Constant selection coefficients													
			PB2	PB1	PA		HA					NP				MP
			G865T	G1862A	T1680C	G2018T	C516A	G603A	C891T	G1258A	χ_516,1258_	A219G	C327T	G868A	C1035T	A394C
Neutral	−22.8	45.5														
**S1**	**−16.1**	**41.2**	**0.4**													
**Neutral**	**−63.9**	**127.8**														
S1	−60.6	130.4		0.2												
Neutral	−965.5	1931.0														
**S1**	**−932.8**	**1875.9**			**−0.1**											
S2	−931.9	1884.4			−0.1	0.1										
Neutral	−1682.02	3364.0														
S1	−732.8	1476.2								−1.0						
S2	−700.4	1421.7					−0.4			−0.8						
S2E	−675.0	1381.5					−0.4			−0.6	−25.6*					
S3E	−666.6	1375.0					−0.4		0.4	−0.6	−25.4*					
**S4E**	**−659.6**	**1371.6**					**−0.4**	**0.4**	**0.3**	**−0.7**	−26.4*					
Neutral	−1821.7	3643.5														
S1	−1739.0	3490.0												0.5		
S2	−1682.0	3388.1												1.0	1.1	
**S3**	**−1651.0**	**3338.3**										**−0.1**		**0.9**	**1.2**	
S4	−1647.9	3344.2										0.2	−0.1	0.9	1.2	
**Neutral**	**−147.3**	**294.5**														
S1	−147.3	305.6														−0.0

### Multi-gene, multi-locus model

#### Inferences of selection

Data were further evaluated using a multi-segment, multi-locus (MGML) model, which allows for the modelling of linkage disequilibrium between loci on different segments. Segment-wide potential haplotypes constructed in the SGML model were joined across segments, constructing multi-segment potential haplotypes. In so far as no short reads spanning viral segments were available, all possible combinations of segment-wide haplotypes were incorporated in this model. Given these haplotypes, a model of mutation and selection was used to fit the data, this time accounting for the potential effects of reassortment in the model. As detailed in the Methods section, our model assumes that reassortment occurs as it would in a well-mixed viral population, such that any two viruses in the population have an equal probability of exchanging genetic material. As such, we refer throughout to an “effective reassortment rate”, describing the extent of reassortment between random viral segments in the host; we note that this value may differ from the absolute rate of reassortment within a single cell. Reassortment was assessed by calculating the overall model likelihood across a finite range of reassortment probabilities *r* = 0, *r* = 1, and *r* = *k* × 10^−*m*^ for *k* ∈ {1, 2, …, 9} and *m* ∈ {1, 2, 3}. All loci identified as potentially being under selection in the SGML model were included in this inference. With the NP segment, while the C372T variant was not identified as being under selection by the SGML model, polymorphisms at this locus were identified in multiple individuals; this locus was also included in the MGML inference.

Under an initial application of the MGML model, in which the effective rate of reassortment between segments was set to zero, selection was inferred to act at a total of four loci across the influenza genome (Table 3). Fitting a consistent fitness landscape to data from all 13 individuals, evidence for selection was inferred in the HA segment against the variants C516A and G1258A, in the NP segment, against the C327T variant, and in the PA segment against the T1680C variant. Evidence for epistatic effects between variants on different segments was found, with the best fitting model inferring epistasis between pairs of alleles on the HA, PA, and NP segments. However, the signal suggesting epistasis between variants within HA was lost. Thus, including interference effects across segments into the model led to a reduction in the number of loci at which selection was inferred.

**Table 3:** Inferred fitness coefficients for the multi-segment, multi-locus model across all individuals given a zero rate of reassortment. Best fitting models are shown for each number of parameters; the best fitting model is highlighted in bold text. The parameter χ denotes an epistatic interaction between alleles.

Model	Likelihood	BIC	Constant selection coefficients								
			HA		NP	PA	PB2				
			C516A	G1258A	C327T	T1680C	G865T	$\chi_{516,1258}^{HA}$	$\chi_{516,327}^{HA,NP}$	$\chi_{1258,1680}^{HA,PA}$	$\chi_{327,1680}^{NP,PA}$
Neutral	−2965.1	5930.1									
S1	−1798.0	3609.1		−1.1							
S2	−1693.7	3413.4	−0.6	−1.0							
S3	−1612.4	3282.0	−0.8	−1.1	−0.4						
S4	−1505.19	3062.6	−1.0	−1.0	−0.6	−0.7					
S4E	−1466.1	2997.4	−0.9	−1.3	−0.9	−0.6					1.5
S4E2	− 1443.2	2964.7	−1.0	−0.9	−0.9	−1.1				−7.8	1.6
**S4E3**	**−1434.4**	**2960.2**	**−1.0**	**−0.9**	**−0.9**	**−1.2**			**−0.4**	**−19.0**	**1.6**
S4E4	−1433.7	2971.8	−1.0	−0.9	−0.9	−1.2		−0.2	−0.4	−19.0	1.6

Of the four variants for which evidence of selection was found, two, in the HA segment, are associated with non-synonymous substitutions. These substitutions have been documented in a previous analysis of these sequence data that focused exclusively on subjects from the first challenge study [15]. The variant at locus 516, representing the amino acid substitution H156Q on the HA1 peptide, has previously been linked with adaptation to the *in ovo* environment [64, 65], while the variant at locus 1258, representing the amino acid substitution G404D (the 75^th^ residue of the HA2 peptide), was identified as decreasing viral stability, such that the variant virus was less immunogenic in a ferret host [66]. As such, within-host evolution in HA appears to be driven by purifying selection for the reversion of passaging adaptation, and a gain in viral stability. Calculations based upon the inferred haplotype frequencies show the increase in the frequency of the reverted HA genotype, in addition to a general decrease in sequence diversity when measured in terms of information entropy; details are given in S12 Fig.

**Fig 3:**
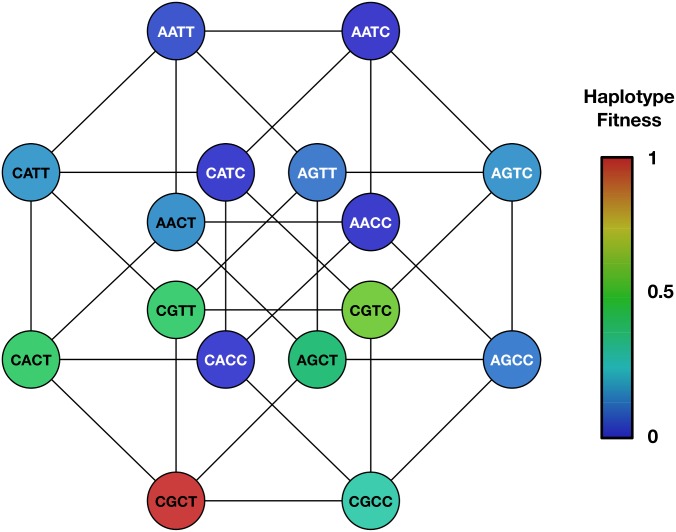
Inferred haplotype fitnesses. Reported haplotypes show the composition of the viral sequence at the nucleotide positions HA 516, HA 1258, NP 327 and PA 1680 respectively. Colour indicates inferred relative fitness from blue (0) to red (1). The haplotypes form a hypercube; lines indicate haplotypes differing by a single nucleotide.

Dividing subjects by the treatment they received, inferences of selection were conducted across subjects receiving either the standard or early treatment regime. Similar results were obtained in each case, with selection being inferred on the four alleles mentioned above in each case. Within individuals under the standard treatment regimen, selection was also inferred to act in favour of the G865T variant in PB2; this variant was detected as being polymorphic in a single subject, in whom it increased in frequency over time. Differences in the inferred epistatic parameters were also evident for each model. However, a comparison of separately- and jointly-inferred models of fitness, calculated using the Bayesian Information Criterion, favoured a model of consistent selection acting across all individuals (Table 4); this model is represented in Fig 3. As may be observed from the figure, the inferred landscape has a single peak, representing the consensus sequence of the virus before passaging, with variants that increased in frequency during passaging incurring a fitness cost when the virus is returned to a human host. In general, as further mutations away from the consensus sequence are accumulated, viral fitness decreases, with the exception of the variants in NP and PA, between which positive epistasis was inferred; here positive epistasis implies that each variant imparts a fitness benefit to the virus in the presence of the other, offsetting the inherent cost of each variant.

**Table 4:** Inferred parameters and likelihoods for all subjects, and for subjects split by treatment, given a zero rate of reassortment.

Dataset	Likelihood	BIC	Selection parameters										
			HA		NP	PA	PB2						
			C516A	G1258A	C327T	T1680C	G865T	$\chi_{516,1258}^{HA}$	$\chi_{516,1680}^{HA,PA}$	$\chi_{516,327}^{HA,NP}$	$\chi_{1258,327}^{HA,NP}$	$\chi_{1258,1680}^{HA,PA}$	$\chi_{327,1680}^{NP,PA}$
**All**	**−1434.4**	**2960.2**	−1.0	−0.9	−0.9	−1.2				−0.4		−19.0	1.6
Early	−520.4		−0.5	−0.4	−1.0	−1.2			−1.5		−21.6		2.1
Normal	−890.1		−0.8	−1.0	−0.5	−0.6	0.8	−1.2					−1.2
**Total (split models)**	**−1410.5**	**3003.8**											

#### Inferences of effective reassortment rate

**Fig 4:**
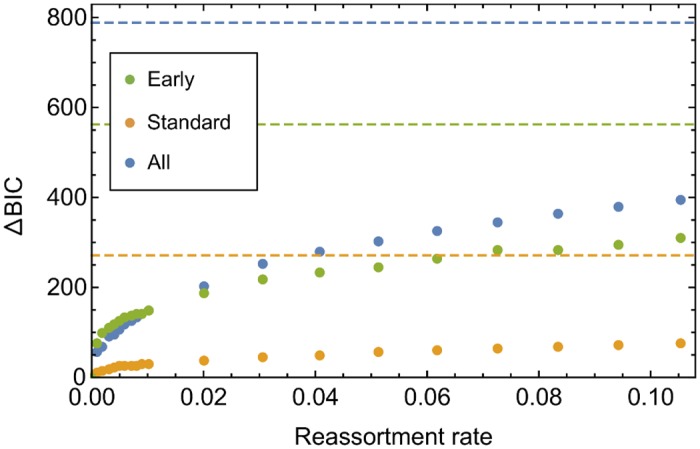
Inferred reassortment rate across all individuals. BIC values from the MGML model, relative to the optimal value, for the early-treatment, standard-treatment, and combined datasets. Horizontal dashed lines indicate the relative BIC value given rapid reassortment between segments. For each dataset, a reassortment rate of zero was inferred, a higher BIC indicating a worse fit to the data. For scale we note that a difference of 10 BIC units indicates strong support in favour of a model.

Repeating the inference of selection at non-zero effective reassortment rates led to different rate-specific inferred fitness landscapes. However, after the optimisation of selection parameters, more rapid reassortment rates gave substantially worse fits to the data such that the best overall fit was obtained at a effective reassortment rate of zero (Fig 4) and with the fitness landscape described above. Considering the dataset as a whole, this model gave a improvement, measured via the Bayesian Information Criterion (BIC), of more than 50 units over a model with reassortment rate of 1 × 10^−3^ gen^−1^, indicating very strong support for a low rate of effective reassortment [67]. Our model implies that linkage between sites on different genome segments had an important role in the observed dynamics of the system.

**Fig 5:**
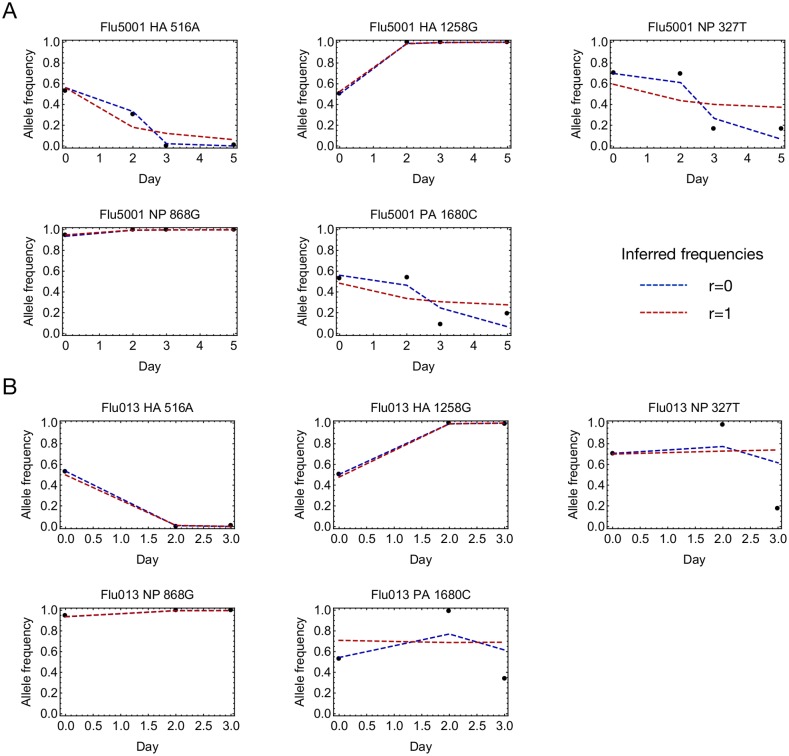
Optimal inferred frequencies at high and low reassortment rates. Viral allele frequencies are shown for the example subjects as black dots. The optimised fit to the data, based on the assumption of a consistent fitness landscape across all subjects, is shown as a red dotted line, for the case of rapid reassortment between segments, and as a blue dotted line for the case of no reassortment between segments. Limiting reassortment between segments allows for the possibility of linkage disequilibrium between alleles on different segments, fitting observed correlations in allele frequencies. Data are shown for all polymorphic alleles at loci included in the model for the subjects **A.** Flu5001 and **B.** Flu013.

Examination of allele frequencies within different segments provided a qualitative understanding of the low inferred rate of reassortment (Fig 5); in some subjects allele frequencies within different segments changed in a non-monotonic and apparently correlated manner (allele frequencies for all trajectories included in the MGML model are shown in S13 Fig). A lack of reassortment would directly explain these observations. Firstly, alleles which are in linkage disequilibrium change frequency in a correlated manner. Secondly, while alleles evolving under constant selection are expected to change frequency in a consistent direction, linkage disequilibrium between alleles can produce non-monotonic change via clonal interference and hitchhiking effects.

**Fig 6:**
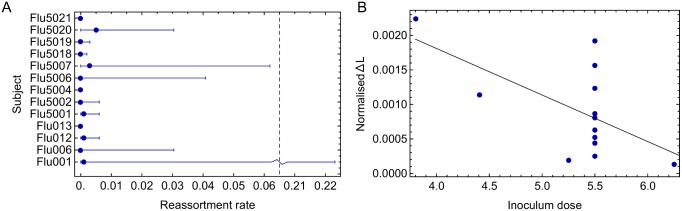
Individual-specific reassortment rates. **A.** Optimal reassortment rates inferred for individual subjects. Error bars, shown as horizontal lines, correspond to confidence intervals obtained via a likelihood ratio test. **B.** Correlation between the extent of evidence for a limited reassortment rate, normalised by the extent of available data, and the size of the inoculum dose. The black line shows a linear regression model fitted to the data.

Subject-specific likelihoods for the rate of reassortment were also recovered from the data. Under the assumption that a consistent fitness landscape applied to the virus across all subjects, non-zero rates of reassortment were sometimes inferred. For example, in the subject Flu5020, the maximum likelihood effective rate of reassortment was calculated to be 0.005 gen^−1^ (Fig 6A). In many cases the magnitude of reassortment could not be determined with high precision.

We note that, in general, high rates of reassortment may be difficult to infer directly from sequence data of the form considered here, in which each read describes alleles only from a single segment, with no multi-locus, multi-segment data. Specifically, a model of limited reassortment can produce a good fit to the behaviour of a system with very rapid reassortment, by fixing the linkage disequilibrium between cross-segment loci to be close to zero. As such, an inference of reassortment rate in a system with rapid reassortment, or in which there is no initial linkage disequilibrium, will produce a flat likelihood curve, with little potential to identify reassortment correctly. By contrast, in a system with significant linkage disequilibrium, and where reassortment is slow, there is far greater potential for a distinction to be made between proposed reassortment rates. An illustration of this effect, based upon simulated data, is shown in S14 Fig. In a system with rapid reassortment, inferences conducted using models with high and low reassortment rates give similar results. However, in a system without reassortment, there is a clear preference for the model in which the rate of reassortment is correct. In the real dataset, inferred frequencies for the data from subject Flu001, in whom the uncertainty in the inferred reassortment rate was greatest, show that both low and high reassortment rates produce similar model fits (S15 Fig).

Exploring the idea that the extent to which reassortment can be inferred is indicative of the reassortment rate itself, we obtain a result consistent with previous studies that have reported a link between the size of the inoculum dose used in experimental influenza infections, and the extent of reassortment between segments [32]. We consider as a proxy for the extent of recombination the magnitude of change in the subject likelihood function between the optimal value and that obtained for a model of rapid reassortment. While this ‘flatness’ of the likelihood function is determined both by the initial extent of linkage disequilibrium between alleles and the reassortment rate, all subjects were inoculated from the same viral stock, such that the former parameter is likely to be relatively consistent between infections. Given that doses were varied in only one of the two challenge studies, our ability to identify a link is somewhat limited. Nevertheless, as shown in Fig 6B, an increased inoculum dose was associated with a decreased likelihood difference between models (p = 0.028), suggestive of a positive relationship between reassortment rate and inoculum dose.

#### Validation against simulated data

The ability of our approach to infer selection and reassortment parameters were validated against simulation. A low reassortment rate was successfully inferred, with variance between the inferred selection parameters being consistent with previous simulation studies [42]; in some cases it is possible for sets of selection parameters which contain compensatory errors to give a good fit to the correct relative haplotype fitness values. More details are given in S1 Text and S16–S18 Figs.

## Discussion

We have applied a novel multi-segment model of within-host evolution to data from infections in multiple individuals during two influenza challenge studies. Our approach for inferring selection is conservative with regard to the quantity of information contained within next-generation sequencing data, and in preferring simpler explanations of variant frequency change. Using our approach, we infer the key evolutionary effects influencing the adaptation of the within-host population in the human subjects under study.

Building upon previous approaches for inferring selection from influenza populations [41, 42, 61], our method infers selection not only at the level of events occurring at a single locus, or within a single segment, but across the entire influenza genome. As inferences were conducted across greater numbers of loci, in the SGML and MGML models, a decrease was observed in the number of loci at which selection was inferred. While the single locus test identifies potential non-neutrality at 16 loci over 6 gene segments, under the MGML model, selection was inferred to apply at only four of these positions within three gene segments. Two reasons likely underlie this reduction in the apparent complexity of the selective model. Firstly, due to linkage disequilibrium, selection acting at one locus can cause changes in the frequency of alleles at multiple other loci; inter-segment associations between alleles may cause changes in the frequencies of alleles not directly under selection. Accounting for linkage disequilibrium between alleles reduces false positive inferences of selection caused in this manner. Secondly, as more data are brought into the model, the model penalty for adding another parameter for selection increases. Where a polymorphism is observed in only a single individual, evidence of genuine selection may not be sufficient to be detected by our model, leading to false negative calls of selection. As such, our inferences describe the core set of fitness effects shaping the evolution of the virus, rather than a comprehensive fitness landscape.

Across all subjects, we infer the key driver of evolution to be the reversion of mutations in HA acquired during passaging before inoculation into individual patients. However, selection was further inferred for variants in the NP and PA segments. The time of administration of oseltamivir to patients did not have a statistically significant effect upon the inferred fitness landscape. Although the emergence of oseltamivir resistance has been documented as having arisen during a single infection [68], in this case no evidence of drug resistance was observed; with regard to the genetic composition of the virus, drug therapy had no apparent impact. While early oseltamivir treatment led to reductions in the severity of symptoms and the length of infection [69], it did not have a distinct evolutionary effect on the viral genome.

Among the variants identified as being under selection, those occuring in HA could be attributed to the reversion of mutations acquired during passaging. The variants C327T in NP and T1680C in PA are both synonymous in nature, and identifying a biological rationale for selection acting upon them requires a little more speculation. Both variants were called as polymorphisms in all subjects, though the explanation for how these variants could affect viral fitness is less clear. One possible explanation would be that the variants interfere with a packaging signal within the vRNA. Packaging signals are a necessary component in the selective packaging model for the influenza virus, which asserts that there is some specific element within the primary sequence of the viral RNA ensuring that each of the eight gene segments is incorporated to new virions [70]. While the packaging signals so far identified have tended to be within the UTRs rather than the coding regions of the segments, a few exceptions to this rule exist [71, 72]. However, none of the packaging sequences identified within the coding regions for NP or PA coincide with the loci described here.

Another possible explanation for selection occurring on the synonymous mutations is that these variants interfere with the secondary structure of the viral RNA [73], structure being potentially related to viral fitness due to the need for the RNA to have a specific thermal stability [74]. For example, the synonymous variant C327T in NP identified by this analysis as under selection is located within a region of highly conserved secondary structure [75]. This region, spanning nucleotides 1031–1250 (numbers indicating the (+) RNA), has been characterized as having high stability. As such, this variant (at position 1193 of the (+) RNA), may be neutral or beneficial within the *in ovo* environment, but confer a fitness disadvantage in the human challenge subjects. While secondary structural elements have been identified within the PA segment [76], the regions described have not included the locus 1680, noted here.

In addition to inferring selection for specific variants, we also inferred the presence of epistatic effects acting between alleles in different segments of influenza. Following the discovery that reassortment viruses generated from distinct strains do not reassort in a random manner [77], a considerable body of literature has been developed studying the propensity for gene segments from distinct viruses to be separated, or found together, following a reassortment event. More formal statistical approaches for identifying associations between segments have been developed [78]. Biases in reassortment have been found in human influenza A and B viruses [79, 80], in cell culture [78, 81], and in swine influenza viruses [82]. The focus of this study is somewhat different to these, being concerned with the rate at which effective reassortment takes place rather than what is produced given a reassortment event. Nevertheless, epistasis is an important factor in our study due to the potential for linkage disequilibrium to be maintained between alleles on separate segments via epistatic effects. By accounting for epistasis in our model, we separate the maintenance of linkage disequilibrium caused by epistasis from that arising from a lack of reassortment such that our result cannot be fully explained by non-random reassortment between segments. Our model infers the existence of epistatic interactions between the HA, NP, and PA segments. However, our study is not the ideal vehicle for which to consider such factors; the study of more diverse strains in an environment in which there was rapid effective reassortment would be desirable for making such inferences.

The restricted extent of effective reassortment between segments inferred during the course of infection is perhaps the most surprising result of our study, with a model of zero reassortment granting the best fit to the data. This result was supported by extremely strong model evidence, with a model of low reassortment providing a far better explanation of the data than a model with rapid reassortment. Our model is limited in so far as it requires the polymorphisms in a segment to be observed at significant allele frequencies; as such, our estimates of reassortment were made across a subset of the segments in the influenza genome. Our result should also be understood in proper context; a lower implied within-host reassortment rate does not contradict observations of reassortment events in global influenza populations.

Our study contains a result matching earlier findings in suggesting that the rate of reassortment within a host is linked to the dose received by a single patient. However, our study contrasts with previous work in the extent of reassortment that was observed; while we identify low reassortment rates, an earlier study noted 86% of viruses reassorting during the course of a single infection in a case where a guinea pig was infected with 10^6^ PFU of virus [31]. We suggest that these results may be reconciled under the assumption of a metapopulation model of infection (c.f. [83, 84]) in which interactions between viruses are limited by spatial diversity within the host. Such a model is well-supported by existing knowledge of influenza virus; viruses in cell culture form distinct plaques [85], while genetically distinct influenza populations have been observed at different locations within a single ferret [19], implying that viruses are not uniformly distributed throughout a host. Under such a model, reassortment, as a local process occurring in single cells, will tend to occur between viruses that are genetically more self-similar than viruses drawn randomly from the population; the effective rate of reassortment, as we define it, will be lower than the absolute rate at which segments are swapped between viruses. Assuming a constant absolute rate of reassortment throughout the host, the rate of effective reassortment will be a function of the local genetic diversity of the population, which itself will depend upon the number of distinct viruses founding an infection in any given region of the host respiratory system. As the total number of viruses founding infection in a host increases, more, and therefore statistically more diverse viruses, found each local infection, such that the observed extent of reassortment also increases. This understanding suggests that in a larger host, where the respiratory system occupies a larger physical space, more viruses would be required to create the same local genetic diversity, and hence the same observed rate of reassortment; given the same infectious dose, the observed rate of reassortment will be lower.

Our study does not oppose the vital role played by animal experiments in understanding influenza infection. However with specific regard to reassortment, experiments conducted *in vitro* or in small animal experiments may over-estimate the effective reassortment rate that might be expected in a human infection. Multi-segment effects may have a substantial and previously under-appreciated role in the evolutionary dynamics of within-host influenza.

## Methods

### Sequence processing

The SAMFIRE package [63] was used to call time-resolved multi-locus sequence variants from the data. High quality short reads were identified using the criteria of a median PHRED quality score of at least 30, trimming sequences where necessary to obtain this. Single nucleotide polymorphisms (SNPs) were then identified from reported nucleotides with PHRED score at least 30, for which at least 10 reads, and at least 2% of the reads in a sample, reported the variant. To account for possible PCR bias, variant alleles were only considered if they were identified in at least two samples from the dataset. SNP trajectories, describing the frequency of each SNP over time, were calculated.

In order to quantify the influence of selection, a conservative estimate was made of the extent of noise in the reported sequence data. Under the assumption that changes in allele frequency over time resulted purely from noise, the extent of noise was characterized within the framework of a Dirichlet multinomial model. Where in a given individual *i*, $n_{i}^{a}\left(t\right)$ is the number of copies of a variant allele observed at the polymorphic locus *a* at time *t*, and $N_{a}^{i}\left(t\right)$ is the total number of alleles observed in this individual at this locus at time *t*, we calculated the mean allele frequency across all reads

$${\hat{q}}_{a}^{i}=\frac{\sum_{t}n_{a}^{i}\left(t\right)}{\sum_{t}N_{a}^{i}\left(t\right)}$$ (1)

for each allele. Assuming that observed changes in allele frequency occur only through noise, we then optimised the parameter *C* to maximise the likelihood

$$L=\sum_{a,i,t}L^{D}\left(C,\left\{n_{a}^{i}\left(t\right)\right\},\left\{{\hat{q}}_{a}^{i}\right\}\right)$$ (2)

where $L^{D}$ is the Dirichlet multinomial distribution with parameters $\alpha_{i}=Cq_{a}^{i}$. This approach produces a conservative estimate of the extent of noise in the system, characterised by *C*.

### Single-locus (SL) model

Given an estimate of the extent of noise in the sequenced data, a likelihood model was used to identify potentially non-neutral alleles across the influenza genome using a single-locus model of selection similar to those applied in earlier studies of viral evolution [61]. Here, for data from allele *a* within individual *i*, we generate a set of model allele frequencies $q_{a}^{i}\left(t\right)$, calculating the model likelihood as

$$L^{m}=\sum_{t}L^{D}\left(C,\left\{n_{a}^{i}\left(t\right)\right\},\left\{q_{a}^{i}\left(t\right)\right\}\right)$$ (3)

and comparing models using the Bayesian Information Criterion (BIC) for model selection [86]; under this comparison, a difference of 10 in BIC values indicates strong evidence for the more complex model [67]. In fitting a model to data, our model assumes a “generation time”, representing a round of intracellular reproduction, as occurring in 12 hours [87]. Fitted models describe the deterministic evolution of an allele frequency under either a neutral model, or a model of selection for the allele in question. In order to account for potential hitchhiking effects, we also examined a time-dependent model of selection, in which a different magnitude of selection was used to model selection between each pair of samples within an individual.

Neutral model:

$$q_{a}\left(t\right)=q\;\;\forall t$$ (4)

Constant selection:

$$q_{a}\left(t\right)=\frac{q_{a}\left(0\right)\exp\left(\sigma t\right)}{1-q_{a}\left(0\right)+q_{a}\left(0\right)\exp\left(\sigma t\right)}$$ (5)

Time-dependent selection:

$$q_{a}\left(t\right)=\frac{q_{a}\left(t-1\right)\exp\left(\sigma_{t}\right)}{1-q_{a}\left(t-1\right)+q_{a}\left(t-1\right)\exp\left(\sigma_{t}\right)}$$ (6)

At the single-locus level, time-dependent selection was considered so as to identify significant non-monotonic changes in allele frequency, as might occur via linkage disequilibrium between alleles; in further models where linkage disequilibrium between alleles was explicit to the model, only models of constant selection were considered. As in the models described below, fitness differences between viruses are understood to arise from differences in the respective within-host growth rates of genetically distinct viral strains.

### Single-segment multi-locus (SGML) model

Where more than one variant occurs within a single segment, selection at a single locus may result in changes in the genetic composition of the virus across multiple loci [88]; under such conditions, single-locus measures of selection can produce misleading results [89]. A multi-locus model was used to account for such effects. Data within each segment were compiled to call multi-locus variants across potentially non-neutral loci. Multi-locus variants were then used to construct a set of potential viral haplotypes for each segment. Our approach to haplotype reconstruction, implemented within the SAMFIRE package, is two-fold by design. Given only single-locus reads, the number of potential haplotypes in a segment is an exponential function of the number of loci at which variation was observed. However, given multi-locus reads, limitations in the diversity of the population may be directly observed. Firstly, therefore, we infer a set of potential segment-wide haplotypes sufficient (though perhaps not necessary) to explain the observed multi-locus data, with no concern for the frequencies at which these haplotypes may or may not exist in the population. Secondly, we fit an evolutionary model describing mutation and selection to the variant data, under the constraint that the population is composed of the identified segment-wide haplotypes in some proportion at each recorded point in time, the frequencies of these haplotypes changing according to the evolutionary models. Mutation was modelled as occurring at constant rate between haplotypes:

$$M:q_{a}\left(t+1\right)=q_{a}\left(t\right)+\mu \sum_{b}\left(q_{b}\left(t\right)-q_{a}\left(t\right)\right)$$ (7)

where *q*_*a*_(*t*) is the frequency of the haplotype *a* in the popluation at time *t*, and the sum is conducted over haplotypes differing from *a* by a single nucleotide. The mutation rate was assumed to be *μ* = 10^−5^ per cite per replication cycle [90]. Selection was modelled to occur deterministically, changing the frequencies of different viral haplotypes according to their fitness.

$$S:q_{a}\left(t+1\right)=\frac{q_{a}\left(t\right)\exp\left(\sigma_{a}\right)}{\sum_{b}q_{b}\left(t\right)\exp\left(\sigma_{b}\right)}$$ (8)

where the sum is conducted over all haploytpes *b*, and the fitness of haplotype *a* is determined by exp(*σ*_*a*_), where

$$\sigma_{a}=\sum_{i}s_{i}+\sum_{i,j}\chi_{i,j}+\sum_{i,j,k}\chi_{i,j,k}+\cdots$$ (9)

is a sum of single- and multi-locus (i.e. epistatic) terms, over variant nucleotides *i*, *j*, *k*, …, contained within the haplotype *a*. Hierarchical models of fitness were considered, gradually increasing the number of component terms in the fitness model, and using BIC to select the model giving the best explanation of the observed changes in viral genetic composition [41, 42].

### Multi-segment multi-locus (MGML) model

While reassortment between influenza segments is potentially a rapid process [31], associations have been observed to exist between alleles on different segments [91]. Further to previous modelling approaches, an inference was made of the effect of reassortment upon the viral dynamics, via the use of a multi-segment, multi-locus model. Sites in each segment at which potential non-neutrality was inferred in the multi-locus model were combined, forming potential viral haplotypes spanning all segments at which selection had been inferred. As above, selection and mutation were evaluated across haplotypes, the fitness of any given viral genotype being calculated at the multi-segment haplotype level. However, in addition, reassortment was evaluated between segments, being modelled to occur between haplotypes with probability *r*:

$$R:q_{a}\left(t+1\right)=\left(1-r\right)q_{a}\left(t\right)+r\prod_{g}q_{a,g}\left(t\right)$$ (10)

where *q*_*a*_ now denotes the frequency of viruses with a specific multi-segment haplotype, and *q*_*a*,*g*_(*t*) is the total frequency at time *t* of viruses having the same alleles as *a* across all loci in the segment *g*. The precise mechanism via which influenza segments are packaged into virions is a subject of ongoing research [92]. Following our assumptions of a large, well-mixed influenza population, we here assume a multi-parental model of reassortment, whereby reassortment occurs freely between a proportion of viruses in each generation. Recalculation of statistics under a strict bi-parental model of reassortment reproduced our key inference of a low effective reassortment rate; see S1 Text and S19 Fig. Given a Poisson model, we note that an equivalent reassortment rate *ρ* can be calculated as:

ρ=-log(1-r) (11)

In the above model, reassortment occurs between random genotypes in the population, as would occur if the population was well-mixed. In the same way that the effective population size is used in population genetics to seek to quantify the extent of genetic drift in terms of an idealised population [93], the statistic we measure in this way describes an effective reassortment rate in the viral population. We note that this effective rate may be significantly lower than the absolute rate of reassortment between viruses, as in a case where reassortment primarily occurs between viruses with similar genotypes.

In order to reduce the computational cost of the calculation, the multi-segment model was evaluated only across sites within each individual at which polymorphism was identified; as such, selection for a genetic variant was only considered to affect the evolution within a host if the variant under selection was observed in that host. Polymorphic loci in an individual form a subset of the total set of loci; individual-specific multi-segment haplotypes were constructed as a projection of the complete set of multi-segment haplotypes onto these loci, evaluating the effect of a consistent model of selection on the dynamics of each system.

Calculations involving reassortment rate were conducted across a range of values of *r*. Subject-specific estimates of reassortment were derived from the component of the likelihood function corresponding to the data from that subject. A further measure quantifying the extent of evidence for limited reassortment in an individual subject was calculated as the normalised difference between the maximum likelihood reassortment model at any value of *r*, and under a model of rapid reassortment.

$$\frac{\max{\left\{L^{r}\right\}}_{r}-L^{\left(r=1\right)}}{N}$$ (12)

where *N* was the total number of datapoints collected from that individual.

### Ethics statement

The details of this human challenge study have been previously described in [15, 49–54]. The procedures performed under this study were in accordance with the Declaration of Helsinki. The protocols followed for this study were approved by the institutional review boards (IRBs) of Duke University Medical Center (Durham, NC), the Space and Naval Warfare Systems Center San Diego (SSD-SD) of the US Department of Defense (Washington, DC), the East London and City Research Ethics Committee 1 (London, UK), and the Independent Western Institutional Review Board (Olympia, WA). All participants enrolled in this challenge study provided written consent, as in accordance with standard IRB protocol.

### Code

Code for the MGML model is available from http://websvc.gen.cam.ac.uk/~cjri2/MGML. The SAMFIRE code contains the single-locus model and is available at https://github.com/cjri/samfire. The SGML code is available as part of a previous publication [42].

## Supporting information

S1 Text Statistical properties of the dataset. Properties of the sequence data. Inferences conducted using simulated data. Model validation.
(PDF)

S1 Fig Length of short reads. Lengths of short reads are reported following processing via the SAMFIRE software package. During processing, reads of shorter than 30 nucleotides were removed from the dataset. Where paired-end data were available, paired-end reads were joined into single continuous reads. The length of a short read is then defined as the total distance in the genome between the first and last reported nucleotides.
(TIF)

S2 Fig Genome-wide read depths. Read depths are reported following filtering via the SAMFIRE software package. During processing, nucleotide calls with PHRED score less than 30 were removed; only high-quality data are here reported. Across the 39 samples collected, including the sampling of the innoculum, data are shown for the minimum (blue), median (yellow) and maximum (green) read depths for each genome position.
(TIF)

S3 Fig Alelle frequency trajectories of polymorphisms in the PB2 gene segment. Observed allele frequency values are colour-coded by individual.
(TIF)

S4 Fig Alelle frequency trajectories of polymorphisms in the PB1 gene segment. Observed allele frequency values are colour-coded by individual.
(TIF)

S5 Fig Alelle frequency trajectories of polymorphisms in the PA gene segment. Observed allele frequency values are colour-coded by individual.
(TIF)

S6 Fig Alelle frequency trajectories of polymorphisms in the HA gene segment. Observed allele frequency values are colour-coded by individual.
(TIF)

S7 Fig Alelle frequency trajectories of polymorphisms in the NP gene segment. Observed allele frequency values are colour-coded by individual.
(TIF)

S8 Fig Alelle frequency trajectories of polymorphisms in the NA gene segment. Observed allele frequency values are colour-coded by individual.
(TIF)

S9 Fig Alelle frequency trajectories of polymorphisms in the MP gene segment. Observed allele frequency values are colour-coded by individual.
(TIF)

S10 Fig Alelle frequency trajectories of polymorphisms in the NS gene segment. Observed allele frequency values are colour-coded by individual.
(TIF)

S11 Fig Single-locus scan for alleles potentially under selection. Bayesian Information Criterion (BIC) differences between the best selected model and the neutral model at each locus in the genome. Differences are reported for loci at which polymorphism was detected. A positive BIC difference indicates a weight of evidence in favour of selection, calculated using a single-locus model, applied to data from a single individual. Loci with a positive BIC difference are highlighted with vertical red dotted lines. Solid red vertical lines show loci at which selection was later identified using a multi-locus model with data from all individuals. Circles denote results from individuals receiving standard treatment; squares denote results from individuals receiving early treatment.
(TIF)

S12 Fig Inference of purifying selection within the genome. **A.** Inferred frequency of individuals within each population having the wild-type consensus alleles at loci 516 and 1258 within the HA segment. Under purifying selection, a return to the consensus over time is inferred. **B.** Inferred sequence entropy calculated across the haplotypes used to model viral evolution within each individual. A general decrease in entropy over time is consistent with the action of purifying selection upon the viral population.
(TIF)

S13 Fig Alelle frequency trajectories of trajectories used in the inference process. Observed allele frequency values are colour-coded by gene segment.
(TIF)

S14 FigInferrability of high and low reassortment rates.Simulated data (dots) and inferences (lines) are shown describing two-locus haplotype frequencies $q_{ij}^{11}$ (blue), $q_{ij}^{10}$ (green), $q_{ij}^{01}$ (yellow), $q_{ij}^{00}$ (orange), and single-locus allele frequencies for systems with reassortment rates *R*^*S*^ inferred using models with reassortment rates *R*^*M*^. Inferences were conducted by fitting single-locus allele frequency data. The maximum log likelihood for the system, *L*, is reported. **(a,b)** Where the reassortment rate of the system is high, models with either high or low reassortment rate provide a good fit to the data. **(c,d)** Where the reassortment rate of the system is low, a model with high reassortment rate may be unable to correctly reproduce the behaviour of the system. Where the model reassortment rate is high, the inferred model may nevertheless have instantaneous linkage disequilibrium at the first time point.(TIF)

S15 Fig Optimal inferred frequencies at high and low reassortment rates. Viral allele frequencies are shown for subject Flu001 as black dots. The optimised fit to the data, based on the assumption of a consistent fitness landscape across all subjects, is shown as a red dotted line, for the case of rapid reassortment between genes, and as a blue dotted line for the case of no reassortment between genes.
(TIF)

S16 Fig BIC values inferred from a simulated population at different model reassortment rates. Inferences were performed at different model reassortment rates for a simulated population based upon parameters inferred from the real population. A model which correctly reproduces the low reassortment rate of the input population was clearly favoured by the inference.
(TIF)

S17 Fig Allele frequencies inferred from a simulated population. Dots show samples of allele frequencies collected from each population. Equivalent lines show model fits to the same data.
(TIF)

S18 Fig Fitness landscape inferred from a simulated population. Reported haplotypes show the composition of the viral sequence at the nucleotide positions HA 516, HA 1258, NP 327 and PA 1680 respectively. Colour indicates inferred relative fitness from blue (0) to red (1). Lines indicate haplotypes accessible via a single mutation.
(TIF)

S19 Fig Inferred reassortment rate across all individuals under a pairwise reassortment model. BIC values from the MGML model, relative to the optimal value, for the combined dataset. The data give a close qualitative fit to the values obtained under a multi-parental model.
(TIF)
