# Supplementary material for: The effective rate of influenza reassortment is limited during human infection
Source: PLoS Pathog. 2017 Feb 7;13(2):e1006203. doi: 10.1371/journal.ppat.1006203 (PMC5315410; doi:10.1371/journal.ppat.1006203)
Supplement: S1 Text — Properties of the sequence data. Inferences conducted using simulated data. Model validation. (PDF) [file ppat.1006203.s001.pdf]

Limited effective reassortment shapes influenza dynamics in a human host

Ashley Sobel Leonard[1], Micah T. McClain[2], Gavin J. D. Smith[3], David E. Wentworth[4,8], Rebecca A. Halpin[4], Xudong Lin[4], Amy Ransier[4], Timothy B. Stockwell[4], Suman R. Das[4], Anthony S. Gilbert[5], Rob Lambkin-Williams[5], Geoffrey S. Ginsburg[2], Christopher W. Woods[2], Katia Koelle[1], Christopher J. R. Illingworth[6,7∗]

1 Department of Biology, Duke University, Durham, USA
2 Duke Center for Applied Genomics and Precision Medicine, Durham, NC, USA
3 Programme in Emerging Infectious Diseases, Duke-NUS Medical School, 8 College Road, 169857, Singapore
4 J. Craig Venter Institute, Rockville, Maryland, USA
5 hVivo PLC, The QMB Innovation Centre, Queen Mary, University of London, 42 New Road, London, UK
6 Department of Genetics, University of Cambridge, Cambridge, UK
7 Department of Applied Maths and Theoretical Physics, Centre for Mathematical Sciences, Wilberforce Road, University of Cambridge, Cambridge, UK
8 Current address: Centers for Disease Control and Prevention, MS-G16, 1600 Clifton Road, Atlanta, GA 30333, United States
∗ **E-mail: Corresponding chris.illingworth@gen.cam.ac.uk**

Supporting Text

Properties of the sequence data

Statistics of the genome sequencing data described in the main text are shown in Supporting Figures S1 and S2. The distribution of read lengths reflects the resequencing of samples using two different technologies, data from these technologies being combined. Sequence depth calculations show the number of variants describing variants at each position which passed filtering, including a minimum PHRED score of 30.

Simulated data

Inference of parameters from a simulated system

Simulated data were used to test the potential of our method to infer selection coefficients and reassortment rate. Simulated data were generated for a system behaving according to parameters similar to those inferred for the real system studied here. A four-locus, two-allele system was constructed evolving according to an additive fitness landscape with single-locus fitness coefficients identical to those inferred for the real system (rounded to 1 decimal place). Assuming that all reads describe only a single locus, the four-locus system contains a total of 16 potential haplotypes. Initial haplotype frequencies were generated according to the inferred initial haplotype frequencies for each patient, calculated across the loci HA 516, HA1258, NP 327, and PA 1680, giving thirteen replicate populations. Each replicate was propagated over a simulated four days of evolution according to a deterministic fitness model, assuming a zero reassortment rate for the system. Sample data were collected after each ‘day’, sampling the allele frequency at each locus via a binomial sampling method to a depth of 100 samples. This simulates the collection of data in a system where no multi-locus reads are available. The sequencing depth of 100 is significantly lower than that achieved in the real dataset, but was chosen to reflect the fact that noise in the real sequencing process reduces the information available from each single read.

Inferences of single-locus selection coefficients and initial haplotype frequencies were made for the system under a variety of probabilities of reassortment (parameter r in the main text), beginning at zero, and increasing in increments of 0.001. In line with the construction of the system, reassortment was assumed to occur between three segments, the first containing the first two loci of the system, with the latter two each in different segments. Noting that data were only collected at the single-locus level, the following likelihood was used to evaluate the system

$$L = \sum_{i=1}^{4} \sum_{j=1}^{13} \sum_{k=1}^{4} \log \binom{N_s}{n^1_{i,j}(t_k)} (q^1_{i,j}(t_k))^{n^1_{i,j}(t_k)} \tag{1}$$

where $n^1_{i,j}(t_k)$ is the number of samples from allele i in individual j with the variant allele at time k and the parameter $q^1_{i,j}(t_k)$ is the sum of the inferred frequencies of the eight potential haplotypes from individual j at time k which have the variant allele at locus i.

Results from the simulation show that a correct reassortment rate can be inferred from allele frequency data alone. Equivalent BIC values calculated from each inference are shown in Figure S16. A clear signal showing an improved fit to the data is evident, supporting the conclusion of a low reassortment rate.

As has been noted previously [1], haplotype fitnesses inferred via our model reflect the overall pattern of the components of the fitness landscape driving the evolution of the system, though greater variance may occur in the individual coefficients from which the haplotype fitnesses are constructed. In our simulation the inferred allele frequencies inferred in a model with zero reassortment rate show a good qualitative fit to the available data (Figure S17). The inferred fitness landscape matches the pattern of the true input landscape (Figure S18), though there was some deviation in inferred single-locus fitness coefficients, with the inferred coefficients (rounded to a single decimal place) of (-1.7, -1.1, -0.2, -1.3) differing slightly from the input coefficients (-1.0, -0.8, -0.9, -1.2).

Inference of reassortment rates given high and low true rates of reassortment

A simpler version of the simulation above was used to illustrate the ability of a model with no reassortment to reproduce the behaviour of a system with rapid reassortment. Here a two-locus, two-allele system was propagated across 10 time-points, sequencing allele frequencies at each time point to a depth of 500. Simulations were conducted both for the case of zero reassortment (equivalently in this case, recombination), and for the case of rapid reassortment.

Given each set of simulated data, inferences were performed, to infer parameters for a model so as to best replicate the behaviour of the simulated system, the fit between the model and the data being calculated using the sum of log binomial likelihoods as above. Inferences were generated using models with either zero, or rapid reassortment, and fitted to data with either zero, or rapid reassortment (Supporting Figure S14).

When the true reassortment rate is high, a small difference between the model likelihoods was observed, showing that it is difficult to correctly identify the reassortment rate in this circumstance. The real data describe a system where the linkage disequilibrium between the two loci is zero, due to rapid reassortment, which is replicated by the model of rapid reassortment. The model with zero reassortment includes some linkage disequilibrium due to the action of selection on the system, but can fit a model in which this amount is very small.

When the true reassortment rate is low, a large difference between the model likelihoods was observed, showing that low reassortment rates can be clearly inferred using our approach. In this circumstance.

while the model with zero reassortment was able to replicate the behaviour of the simulated system, the model with rapid reassortment could not achieve this. Within a model of rapid reassortment, linkage disequilibrium between loci may instantaneously be non-zero, but it is subsequently destroyed by the reassortment process.

Model validation

The ability of our approach to infer selection and reassortment parameters were validated against simulation. The evolutionary parameters inferred for each patient were used to generate a realistic simulated system, from which parameters were re-inferred. A low reassortment rate was successfully inferred. Consistent with previous simulation studies [1], some variance in the inferred selection parameters was observed, but with a generally consistent fitness landscape. Results of the inference from simulation are described in Supporting Text and shown in Supporting Figures S16 to S18. Some understanding of the extent of uncertainty in the inferred selection coefficients can be obtained by comparing results obtained for different groups of individuals (Table 4, Main text). The inference of selection for the variant in PB2 is not made for one dataset as the variant was only identified in a single patient.

Further validation was carried out against a model built under the assumption that reassortment is a strictly bi-parental process. Under this model, the probability of reassortment between two specific multi-segment haplotypes i and j is given by rq_iq_j, the product of the reassortment probability and the two haplotype frequencies. Supposing that a reassortment occurs between two specific haplotypes, segments from the two haplotypes were shuffled, with any resulting combination of segments being equally likely. This model reproduced the result that strong evidence was found for a model of low effective reassortment, as shown in Supporting Figure S19.

Supporting Figures

Figure S1. Length of short reads. Lengths of short reads collected from Illumina (red) and IonTorrent (black) sources. During later processing, reads with fewer than 30 nucleotides were removed from the dataset.

Figure S2. Genome-wide read depths. Read depths are reported following filtering via the SAMFIRE software package. During processing, low quality nucleotide calls were removed; only high-quality data are here reported. Across the 39 samples collected, including the sampling of the innoculum, data are shown for the minimum (blue), median (yellow) and maximum (green) read depths for each genome position.

Figure S3. Alelle frequency trajectories of polymorphisms in the PB2 gene segment Observed allele frequency values are colour-coded by individual.

Figure S4. Alelle frequency trajectories of polymorphisms in the PB1 gene segment Observed allele frequency values are colour-coded by individual.

Figure S5. Alelle frequency trajectories of polymorphisms in the PA gene segment Observed allele frequency values are colour-coded by individual.

Figure S6. Alelle frequency trajectories of polymorphisms in the HA gene segment Observed allele frequency values are colour-coded by individual.

Figure S7. Alelle frequency trajectories of polymorphisms in the NP gene segment Observed allele frequency values are colour-coded by individual.

Figure S8. Alelle frequency trajectories of polymorphisms in the NA gene segment Observed allele frequency values are colour-coded by individual.

Figure S9. Alelle frequency trajectories of polymorphisms in the MP gene segment Observed allele frequency values are colour-coded by individual.

Figure S10. Alelle frequency trajectories of polymorphisms in the NS gene segment Observed allele frequency values are colour-coded by individual.

Figure S11. Single-locus scan for alleles potentially under selection. Bayesian Information Criterion (BIC) differences between the best selected model and the neutral model at each locus in the genome. Differences are reported for loci at which polymorphism was detected. A positive BIC difference indicates a weight of evidence in favour of selection, calculated using a single-locus model, applied to data from a single individual. Loci with a positive BIC difference are highlighted with vertical red dotted lines. Solid red vertical lines show loci at which selection was later identified using a multi-locus model with data from all individuals. Circles denote results from individuals receiving standard treatment; squares denote results from individuals receiving early treatment.

Figure S12. Inference of purifying selection within the genome. A Inferred frequency of individuals within each population having the wild-type consensus alleles at loci 516 and 1258 within the HA segment. Under purifying selection, a return to the consensus over time is inferred. **B** Inferred sequence entropy calculated across the haplotypes used to model viral evoution within each individual. A general decrease in entropy over time is consistent with the action of purifying selection upon the viral population.

Figure S13. Alelle frequency trajectories of trajectories used in the inference process Observed allele frequency values are colour-coded by gene segment.

References

1. Illingworth CJR (2015) Fitness Inference from Short-Read Data: Within-Host Evolution of a Reassortant H5N1 Influenza Virus. Molecular Biology and Evolution 32: 3012–3026.

Figure S14. Inferrability of high and low reassortment rates. Simulated data (dots) and inferences (lines) are shown describing two-locus haplotype frequencies q_{ij}^{11} (blue), q_{ij}^{10} (green), q_{ij}^{01} (yellow), q_{ij}^{00} (orange), and single-locus allele frequencies for systems with reassortment rates R^S inferred using models with reassortment rates R^M. Inferences were conducted by fitting single-locus allele frequency data. The maximum log likelihood for the system, L, is reported. **(a,b)** Where the reassortment rate of the system is high, models with either high or low reassortment rate provide a good fit to the data. **(c,d)** Where the reassortment rate of the system is low, a model with high reassortment rate may be unable to correctly reproduce the behaviour of the system. Where the model reassortment rate is high, the inferred model may nevertheless have instantaneous linkage disequilibrium at the first time point.

Figure S15. Optimal inferred frequencies at high and low reassortment rates. Viral allele frequencies are shown for subject Flu001 as black dots. The optimised fit to the data, based on the assumption of a consistent fitness landscape across all subjects, is shown as a red dotted line, for the case of rapid reassortment between genes, and as a blue dotted line for the case of no reassortment between genes.

Figure S16. BIC values inferred from a simulated population at different model reassortment rates. Inferences were performed at different model reassortment rates for a simulated population based upon parameters inferred from the real population. A model which correctly reproduces the low reassortment rate of the input population was clearly favoured by the inference.

Figure S17. Allele frequencies inferred from a simulated population. Dots show samples of allele frequencies collected from each population. Equivalent lines show model fits to the same data.

Figure S18. Fitness landscape inferred from a simulated population. Reported haplotypes show the composition of the viral sequence at the nucleotide positions HA 516, HA 1258, NP 327 and PA 1680 respectively. Colour indicates inferred relative fitness from blue (0) to red (1). Lines indicate haplotypes accessible via a single mutation.

Figure S19. Inferred reassortment rate across all individuals under a pairwise reassortment model. BIC values from the MGML model, relative to the optimal value, for the combined dataset. The data give a close qualitative fit to the values obtained under a multi-parental model.
