# Supplementary figures and images for: The effective rate of influenza reassortment is limited during human infection
Source: PLoS Pathog. 2017 Feb 7;13(2):e1006203. doi: 10.1371/journal.ppat.1006203 (PMC5315410; doi:10.1371/journal.ppat.1006203)

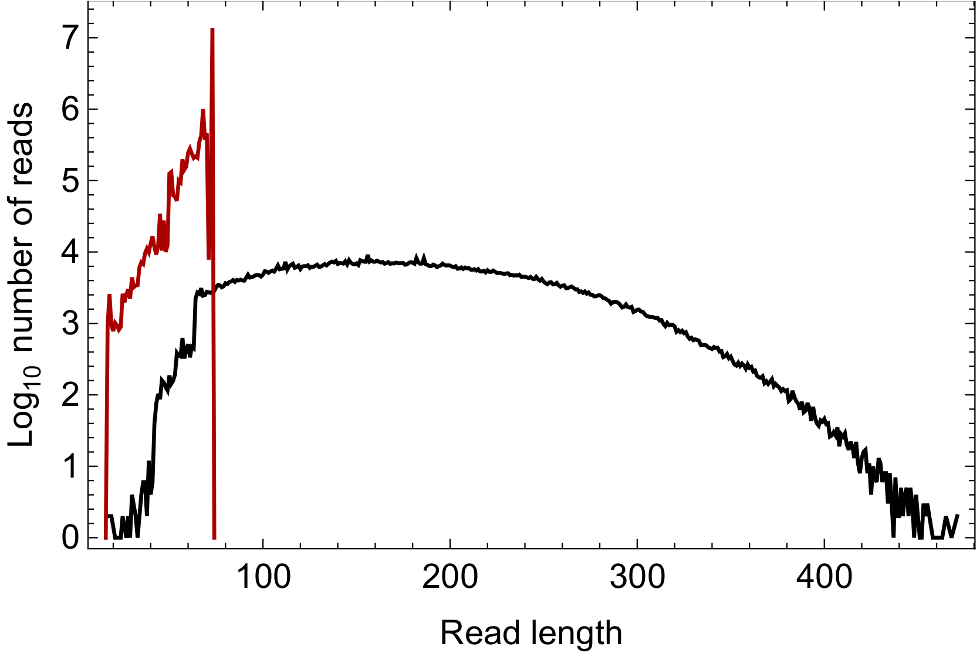

Supplement: S1 Fig — Lengths of short reads are reported following processing via the SAMFIRE software package. During processing, reads of shorter than 30 nucleotides were removed from the dataset. Where paired-end data were available, paired-end reads were joined into single continuous reads. The length of a short read is then defined as the total distance in the genome between the first and last reported nucleotides. (TIF) [file ppat.1006203.s002.tif]

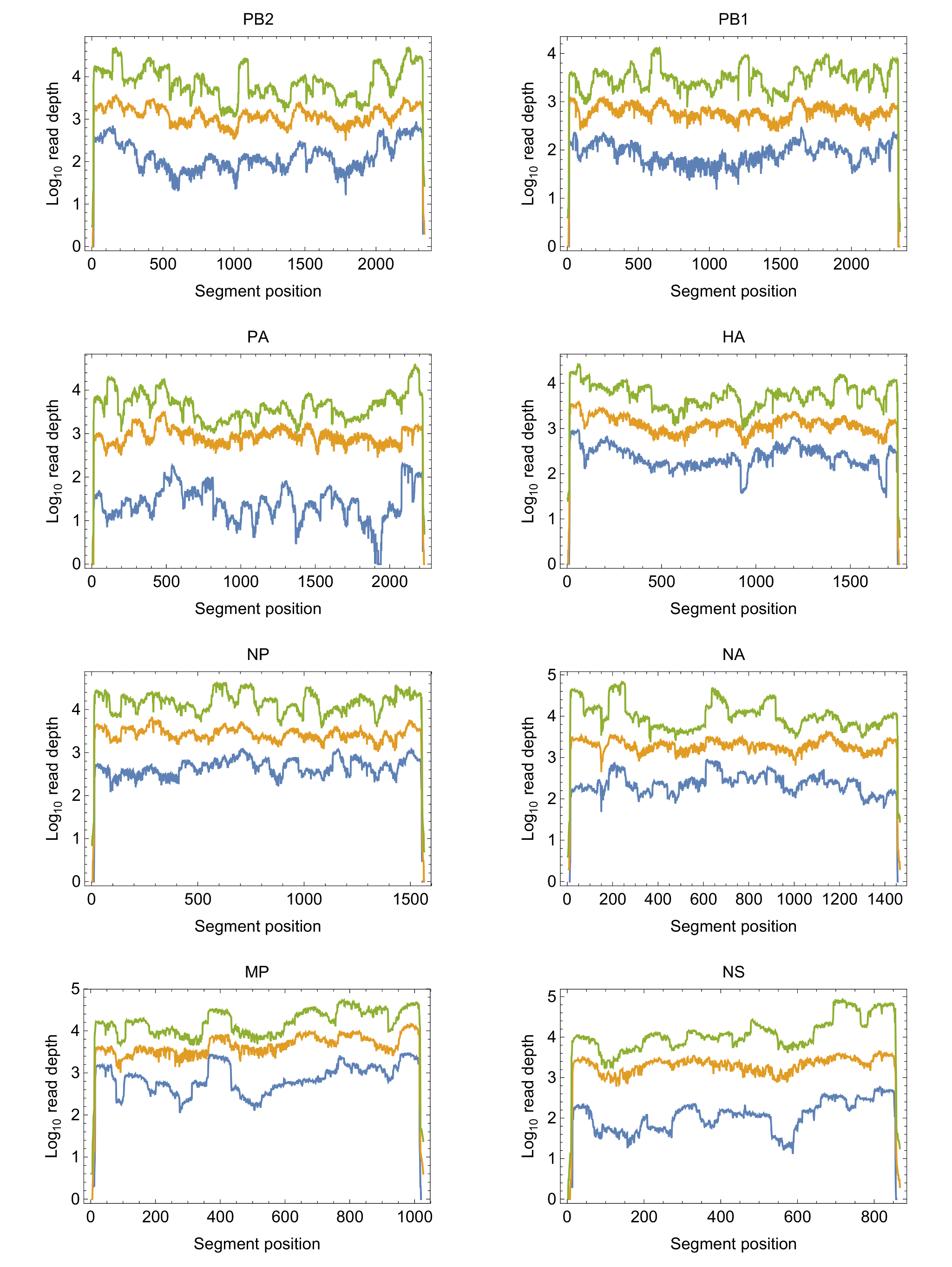

Supplement: S2 Fig — Read depths are reported following filtering via the SAMFIRE software package. During processing, nucleotide calls with PHRED score less than 30 were removed; only high-quality data are here reported. Across the 39 samples collected, including the sampling of the innoculum, data are shown for the minimum (blue), median (yellow) and maximum (green) read depths for each genome position. (TIF) [file ppat.1006203.s003.tif]

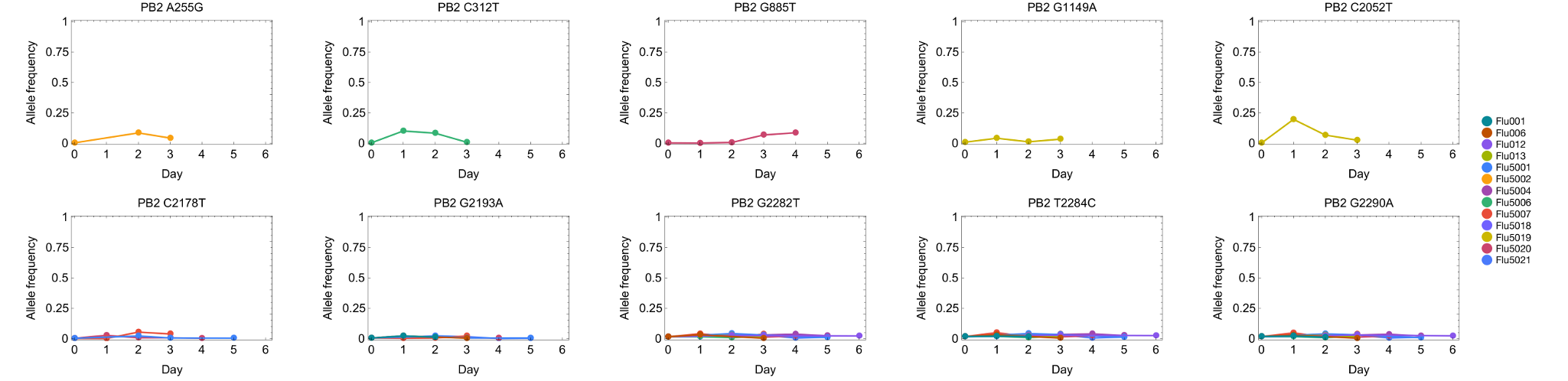

Supplement: S3 Fig — Observed allele frequency values are colour-coded by individual. (TIF) [file ppat.1006203.s004.tif]

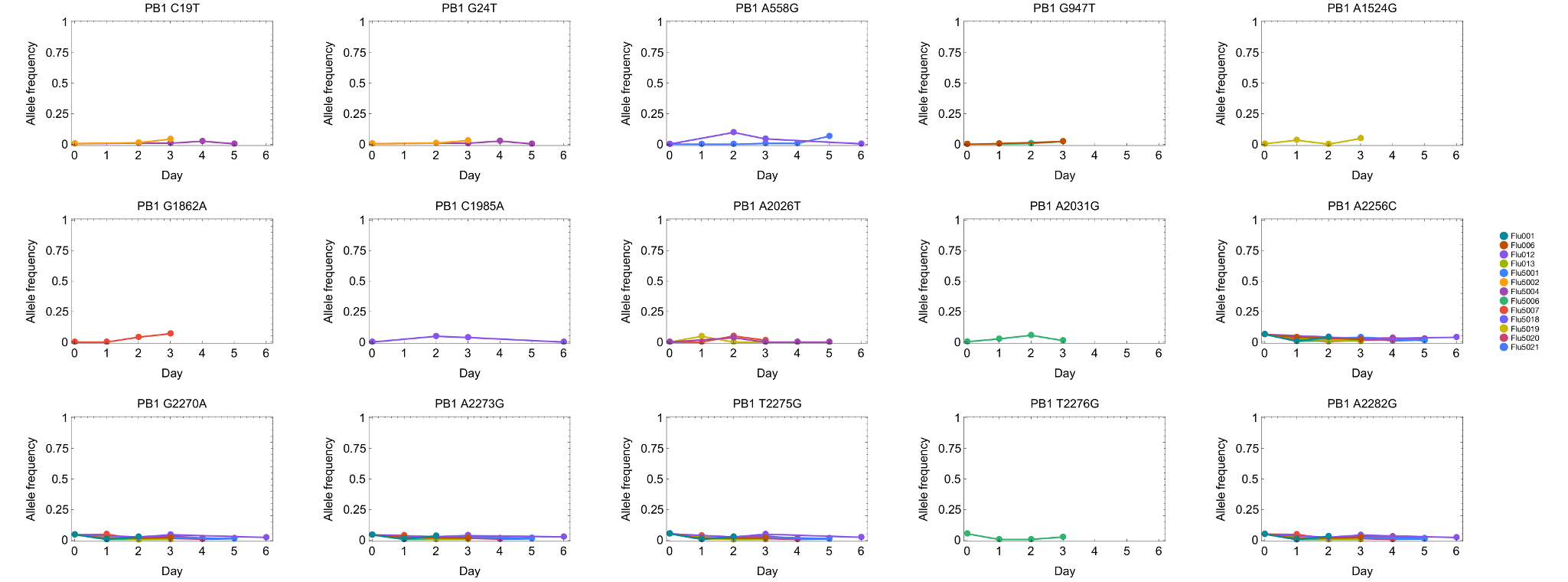

Supplement: S4 Fig — Observed allele frequency values are colour-coded by individual. (TIF) [file ppat.1006203.s005.tif]

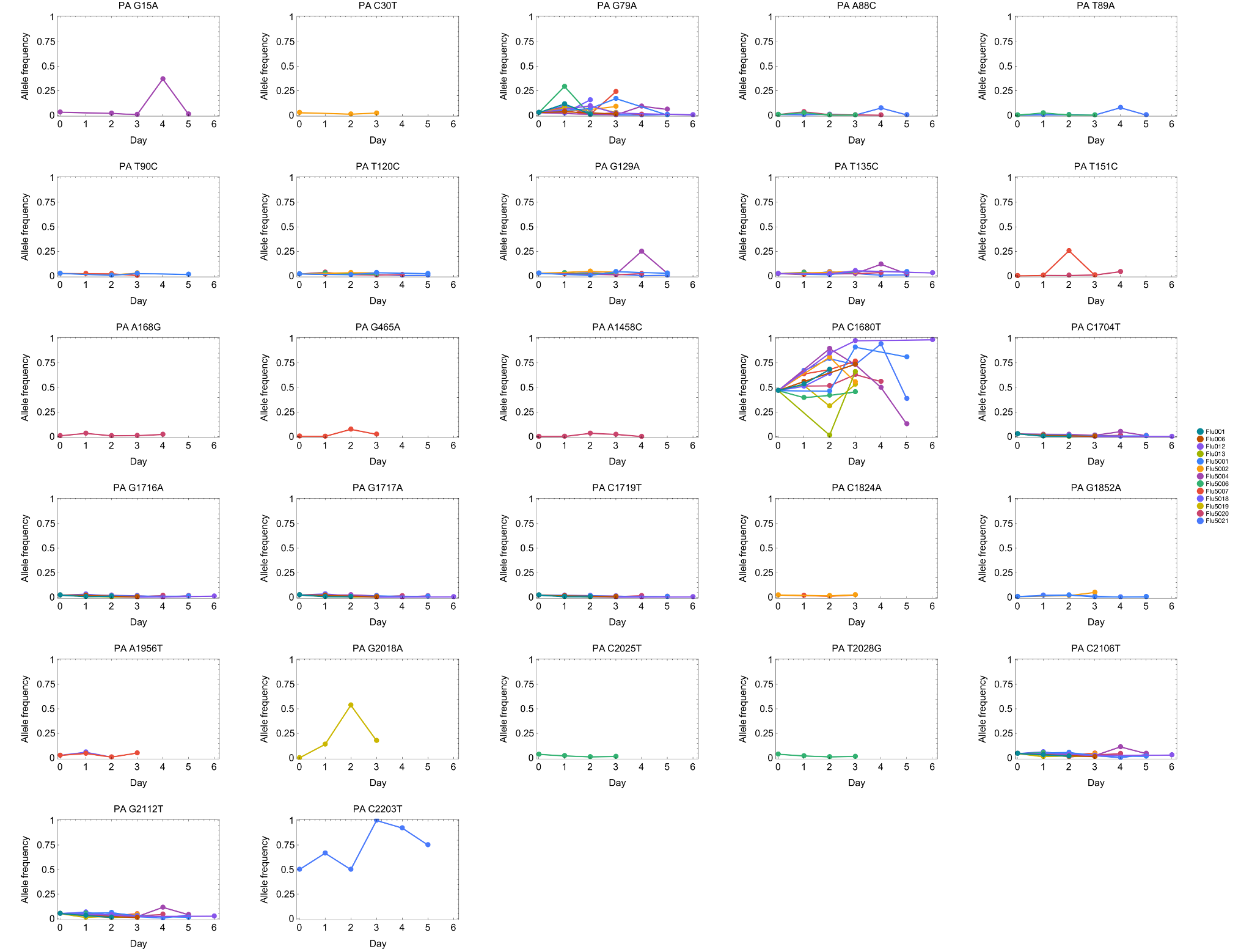

Supplement: S5 Fig — Observed allele frequency values are colour-coded by individual. (TIF) [file ppat.1006203.s006.tif]

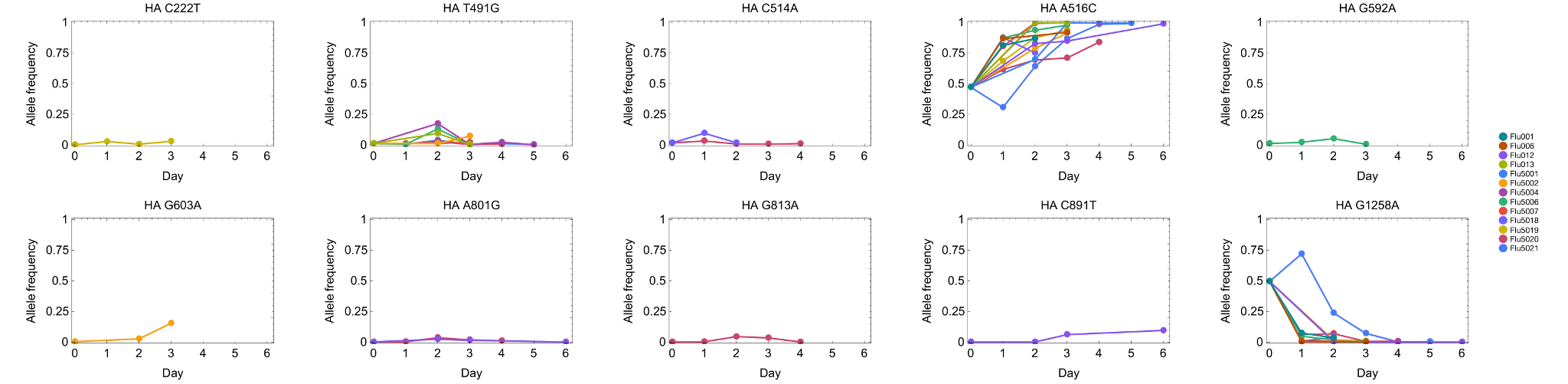

Supplement: S6 Fig — Observed allele frequency values are colour-coded by individual. (TIF) [file ppat.1006203.s007.tif]

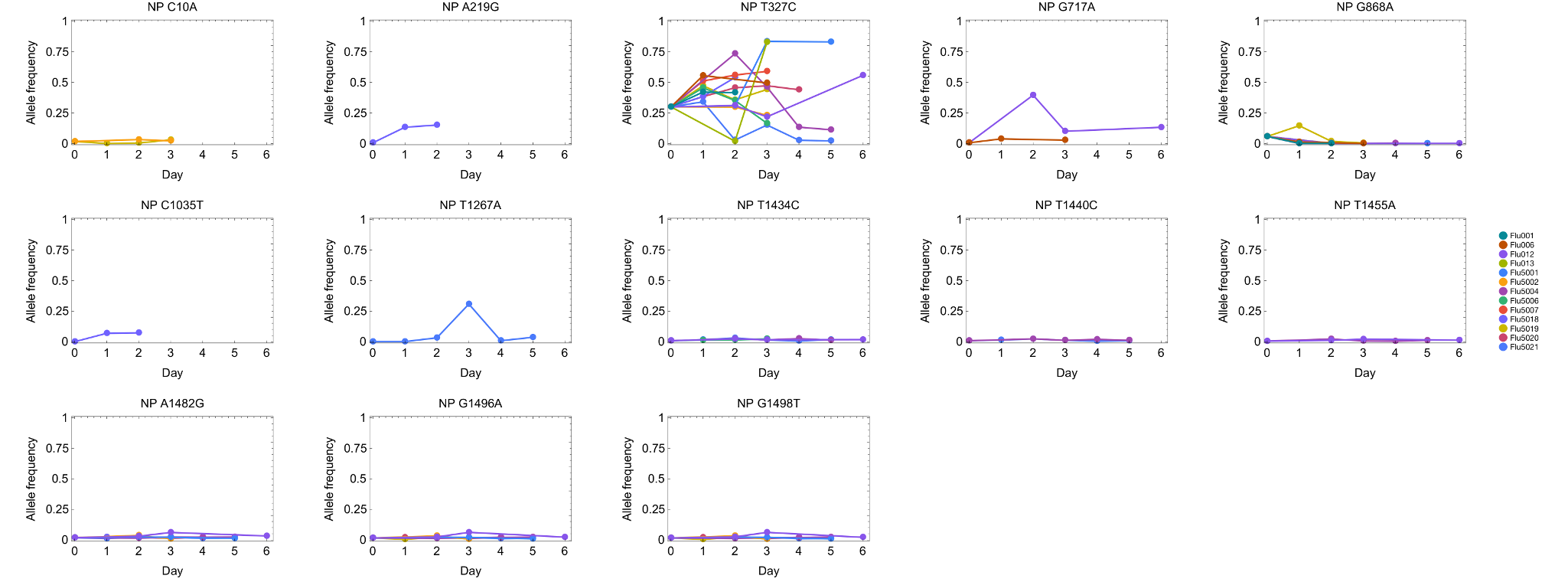

Supplement: S7 Fig — Observed allele frequency values are colour-coded by individual. (TIF) [file ppat.1006203.s008.tif]

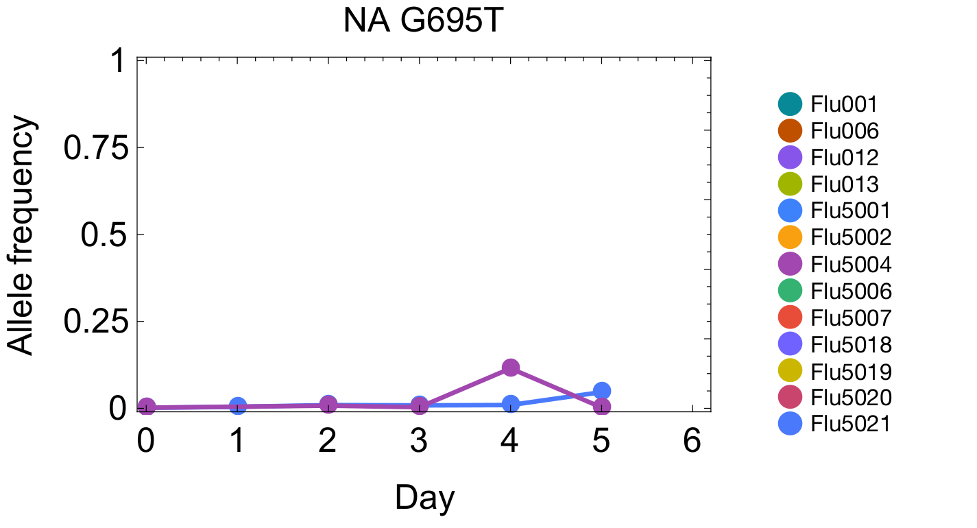

Supplement: S8 Fig — Observed allele frequency values are colour-coded by individual. (TIF) [file ppat.1006203.s009.tif]

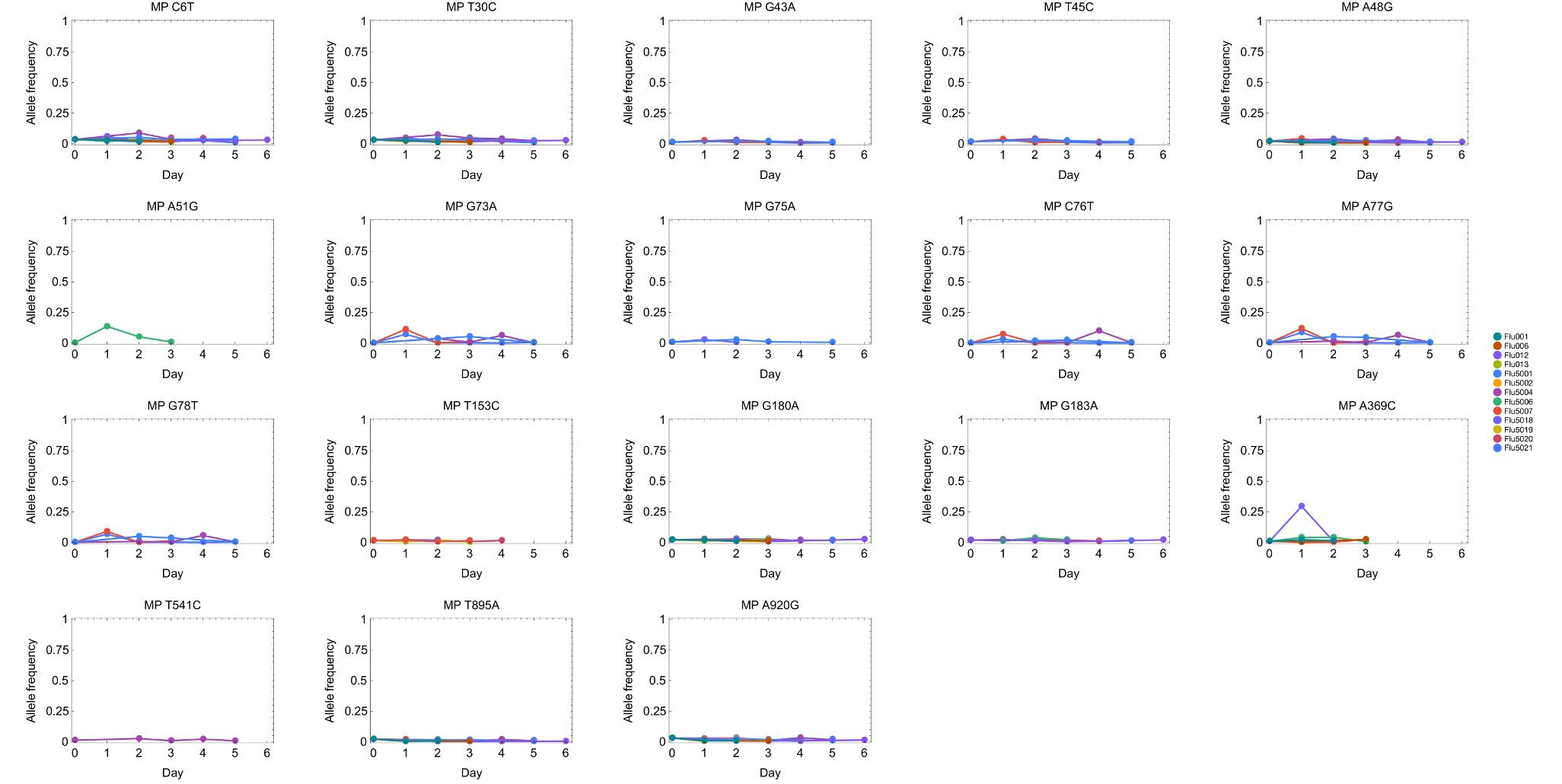

Supplement: S9 Fig — Observed allele frequency values are colour-coded by individual. (TIF) [file ppat.1006203.s010.tif]

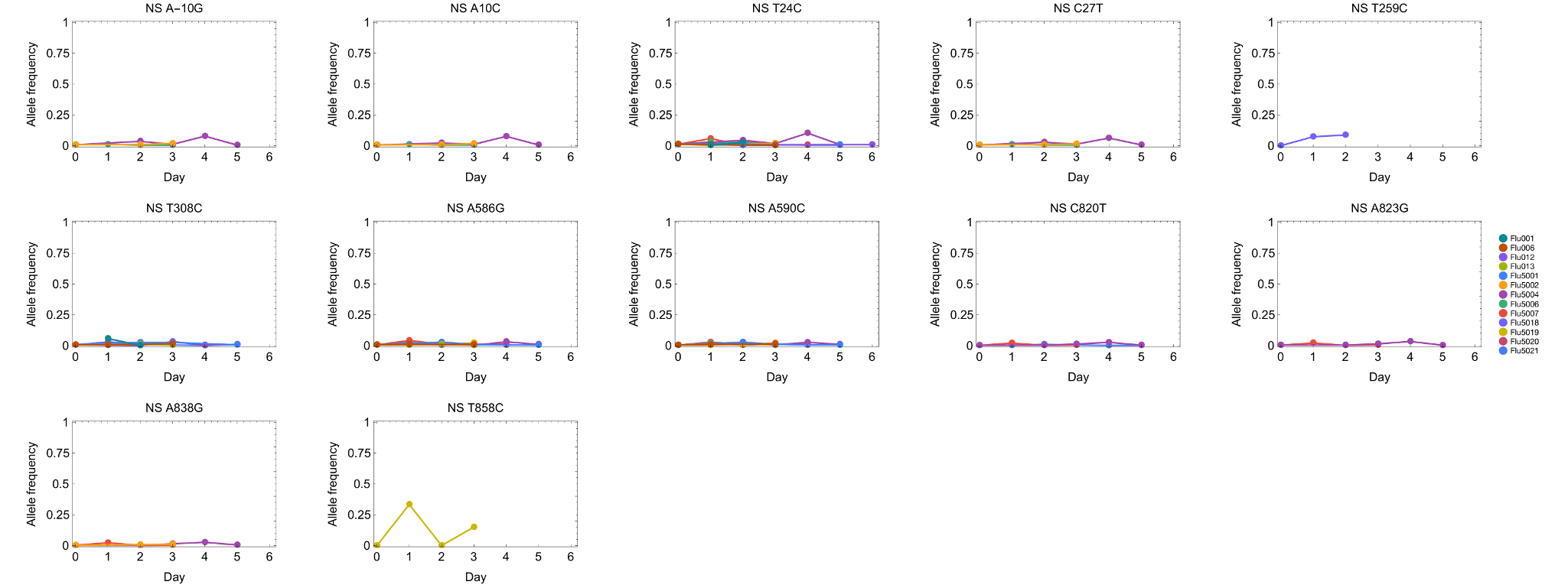

Supplement: S10 Fig — Observed allele frequency values are colour-coded by individual. (TIF) [file ppat.1006203.s011.tif]

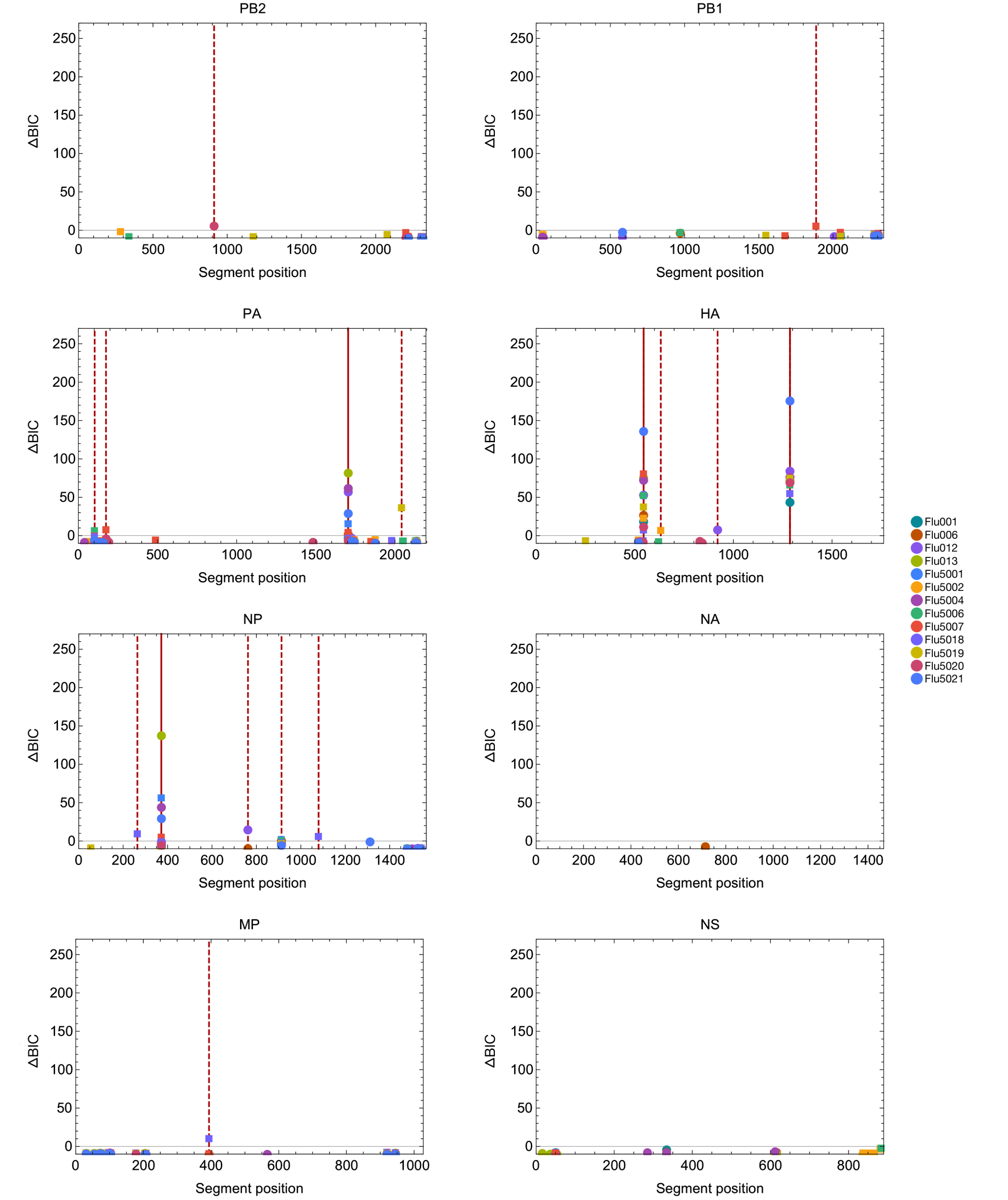

Supplement: S11 Fig — Bayesian Information Criterion (BIC) differences between the best selected model and the neutral model at each locus in the genome. Differences are reported for loci at which polymorphism was detected. A positive BIC difference indicates a weight of evidence in favour of selection, calculated using a single-locus model, applied to data from a single individual. Loci with a positive BIC difference are highlighted with vertical red dotted lines. Solid red vertical lines show loci at which selection was later identified using a multi-locus model with data from all individuals. Circles denote results from individuals receiving standard treatment; squares denote results from individuals receiving early treatment. (TIF) [file ppat.1006203.s012.tif]

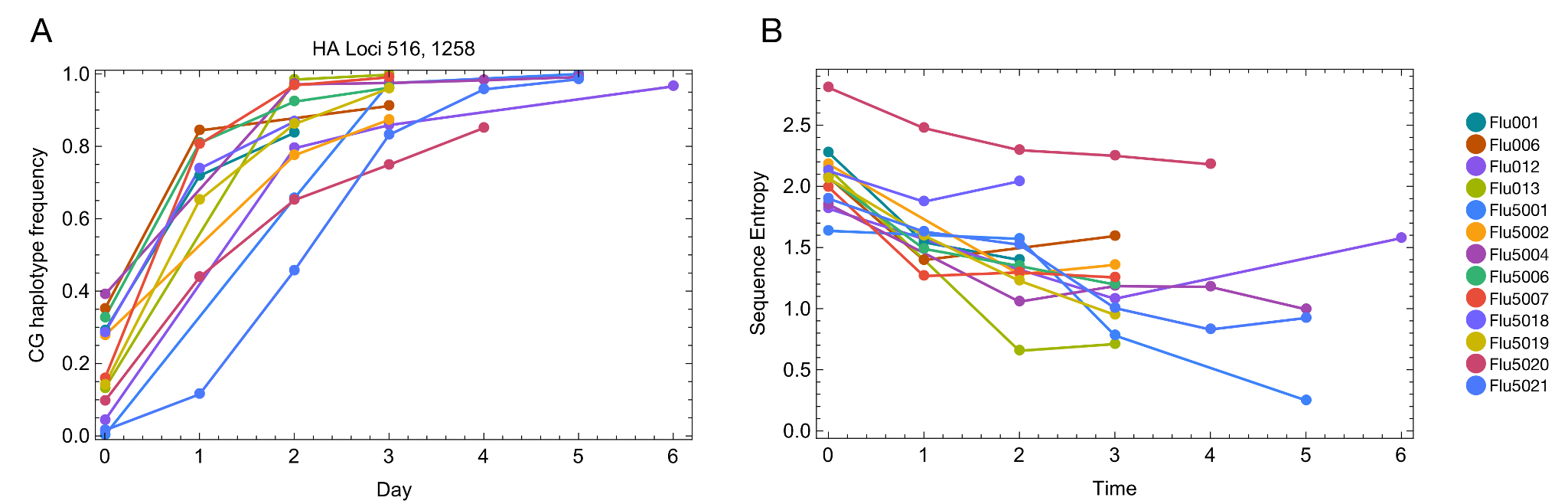

Supplement: S12 Fig — A. Inferred frequency of individuals within each population having the wild-type consensus alleles at loci 516 and 1258 within the HA segment. Under purifying selection, a return to the consensus over time is inferred. B. Inferred sequence entropy calculated across the haplotypes used to model viral evolution within each individual. A general decrease in entropy over time is consistent with the action of purifying selection upon the viral population. (TIF) [file ppat.1006203.s013.tif]

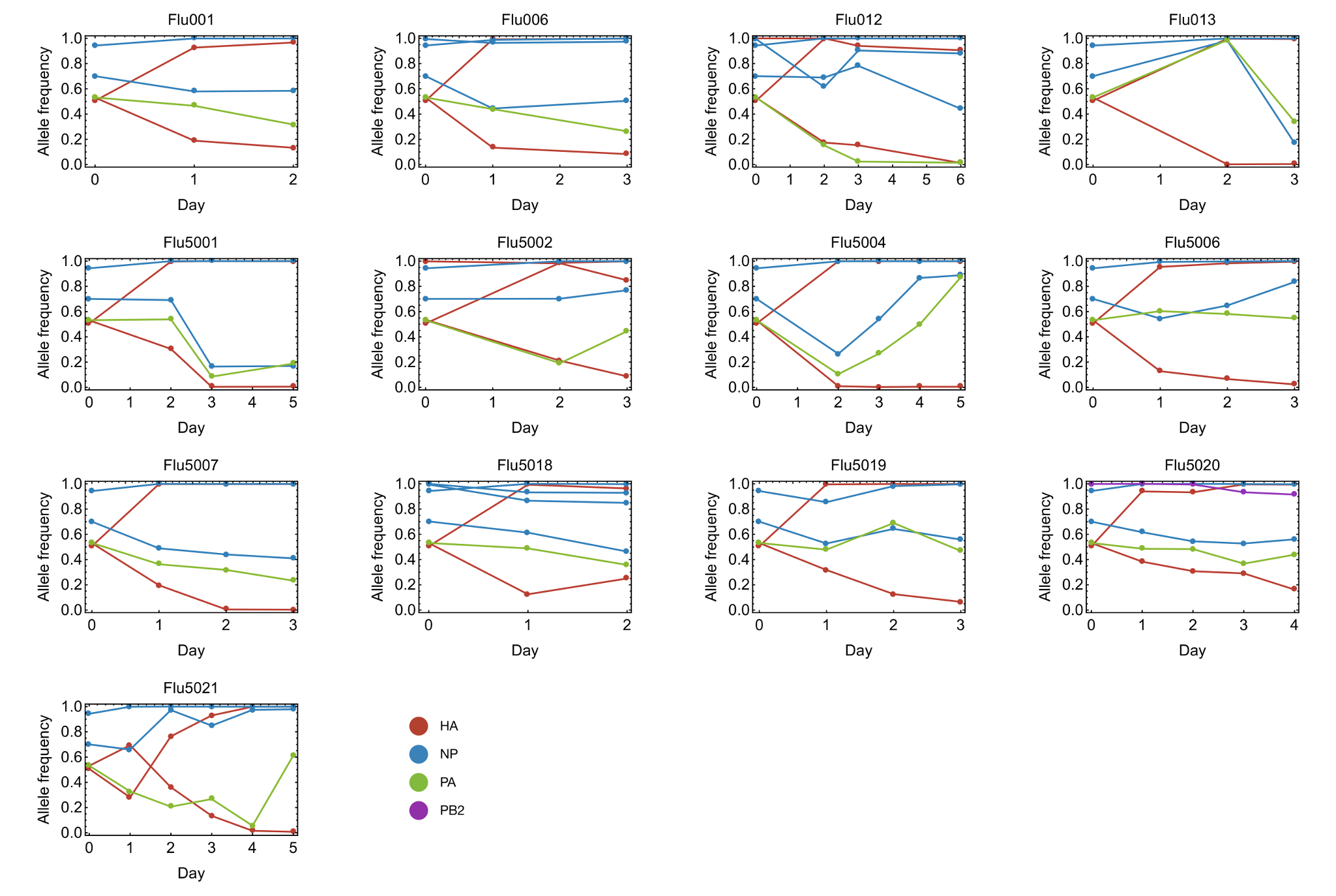

Supplement: S13 Fig — Observed allele frequency values are colour-coded by gene segment. (TIF) [file ppat.1006203.s014.tif]

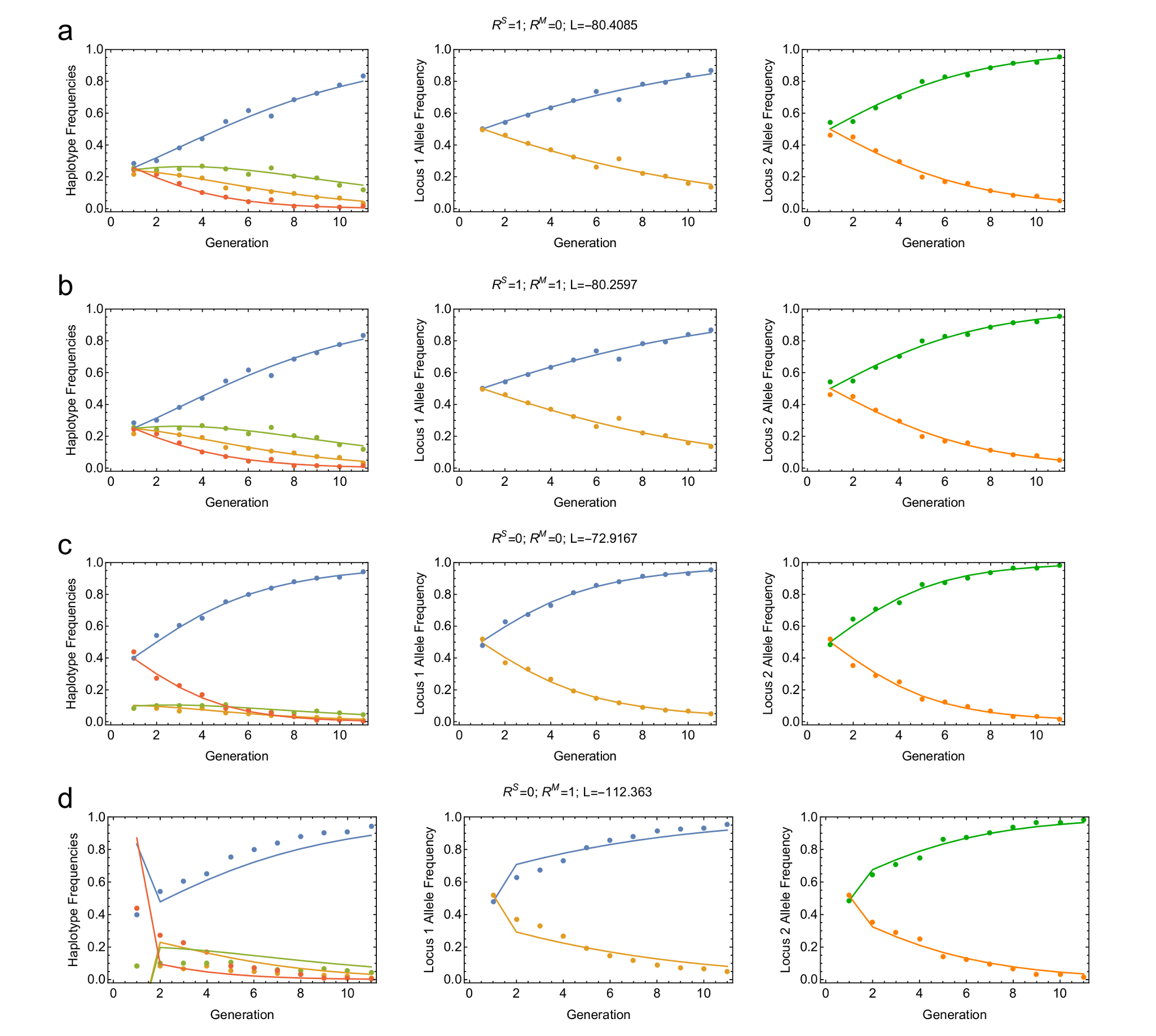

Supplement: S14 Fig — Simulated data (dots) and inferences (lines) are shown describing two-locus haplotype frequencies qij11 (blue), qij10 (green), qij01 (yellow), qij00 (orange), and single-locus allele frequencies for systems with reassortment rates RS inferred using models with reassortment rates RM. Inferences were conducted by fitting single-locus allele frequency data. The maximum log likelihood for the system, L, is reported. (a,b) Where the reassortment rate of the system is high, models with either high or low reassortment rate provide a good fit to the data. (c,d) Where the reassortment rate of the system is low, a model with high reassortment rate may be unable to correctly reproduce the behaviour of the system. Where the model reassortment rate is high, the inferred model may nevertheless have instantaneous linkage disequilibrium at the first time point. (TIF) [file ppat.1006203.s015.tif]

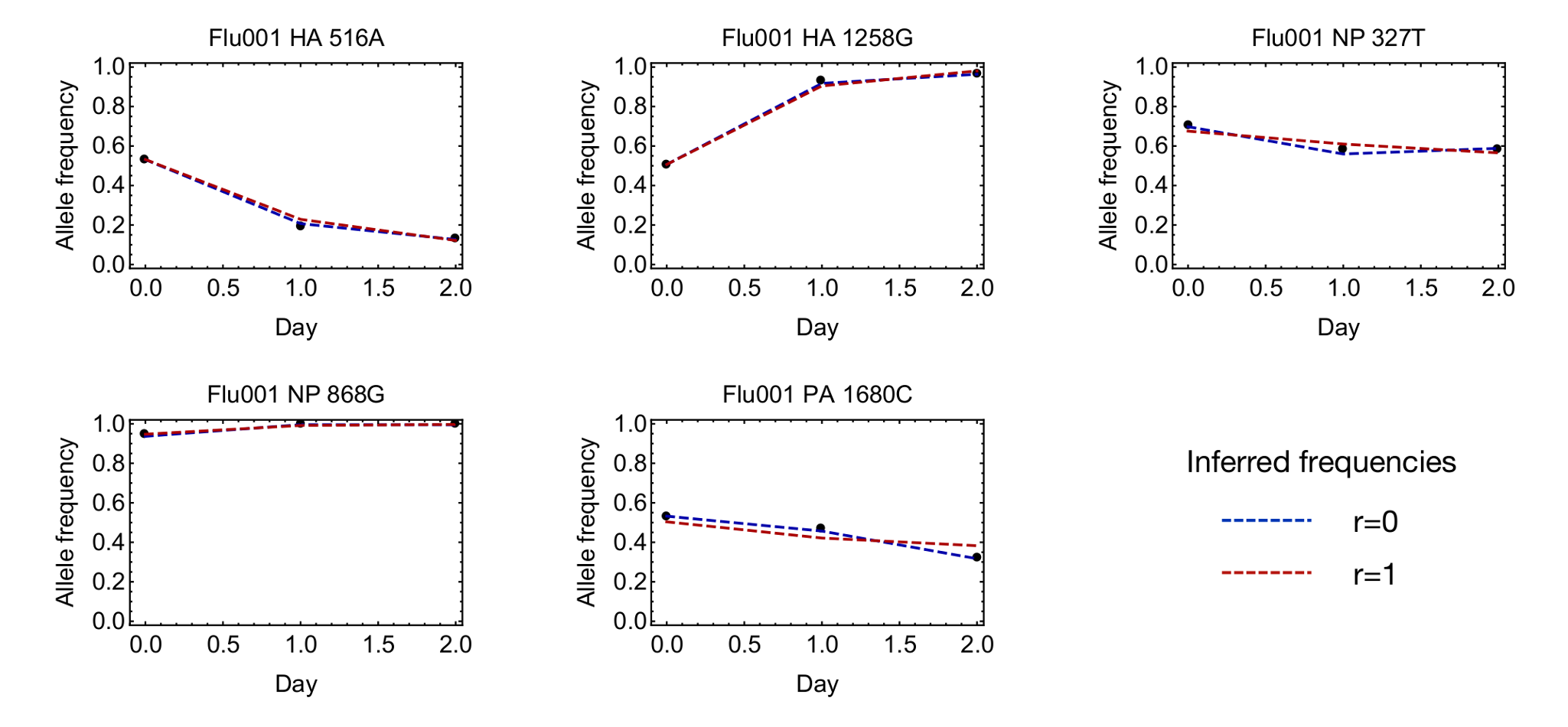

Supplement: S15 Fig — Viral allele frequencies are shown for subject Flu001 as black dots. The optimised fit to the data, based on the assumption of a consistent fitness landscape across all subjects, is shown as a red dotted line, for the case of rapid reassortment between genes, and as a blue dotted line for the case of no reassortment between genes. (TIF) [file ppat.1006203.s016.tif]

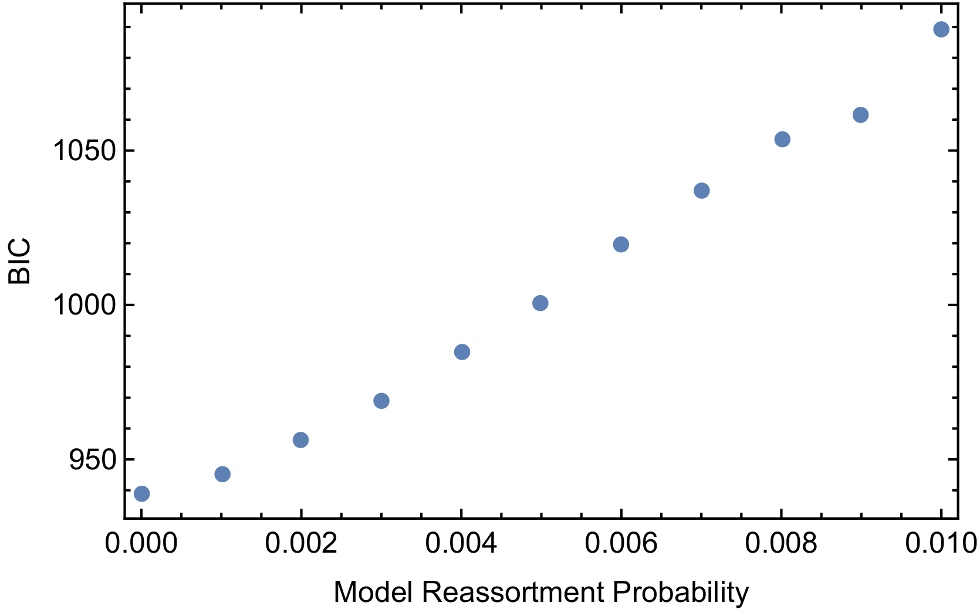

Supplement: S16 Fig — Inferences were performed at different model reassortment rates for a simulated population based upon parameters inferred from the real population. A model which correctly reproduces the low reassortment rate of the input population was clearly favoured by the inference. (TIF) [file ppat.1006203.s017.tif]

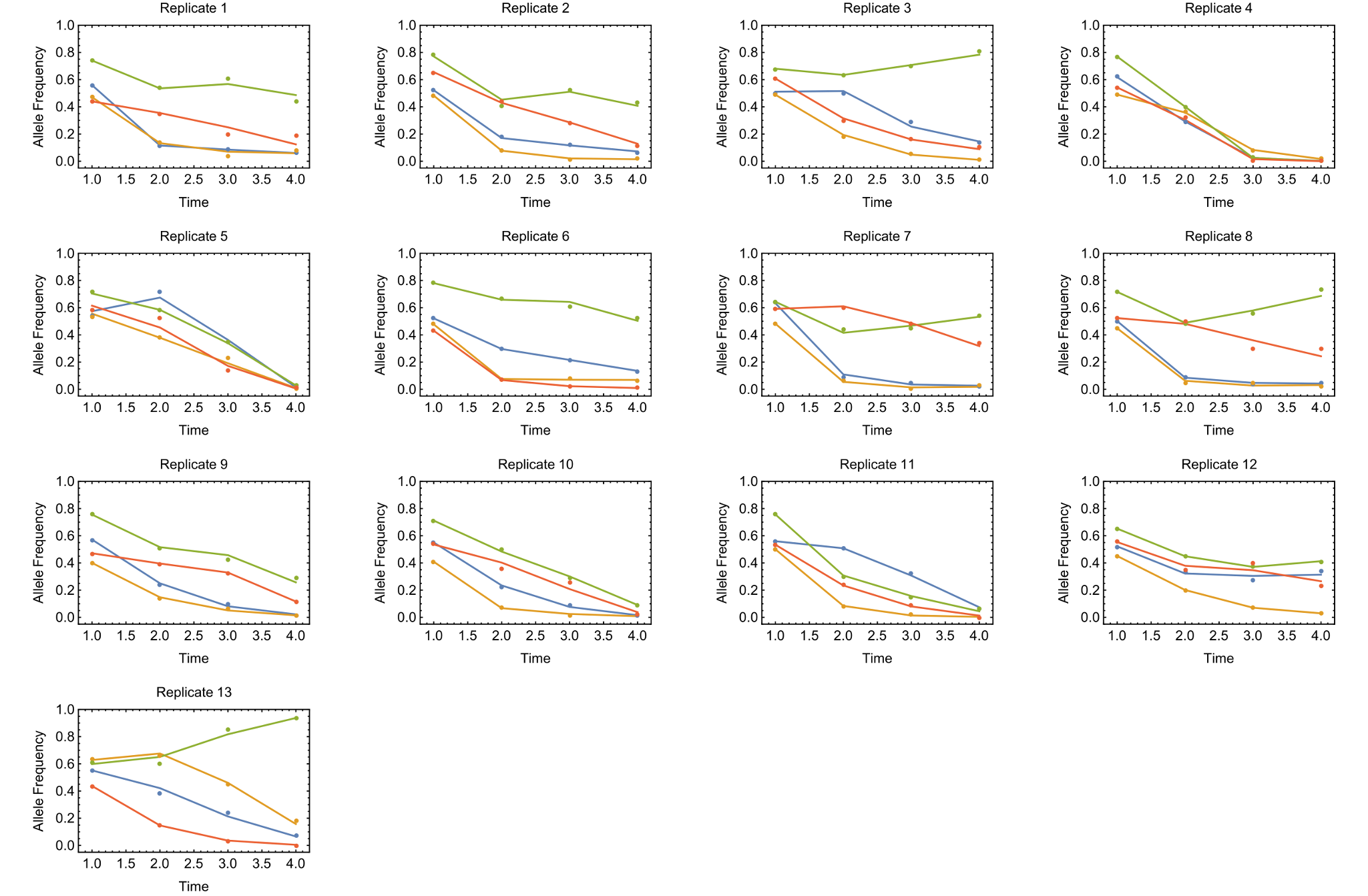

Supplement: S17 Fig — Dots show samples of allele frequencies collected from each population. Equivalent lines show model fits to the same data. (TIF) [file ppat.1006203.s018.tif]

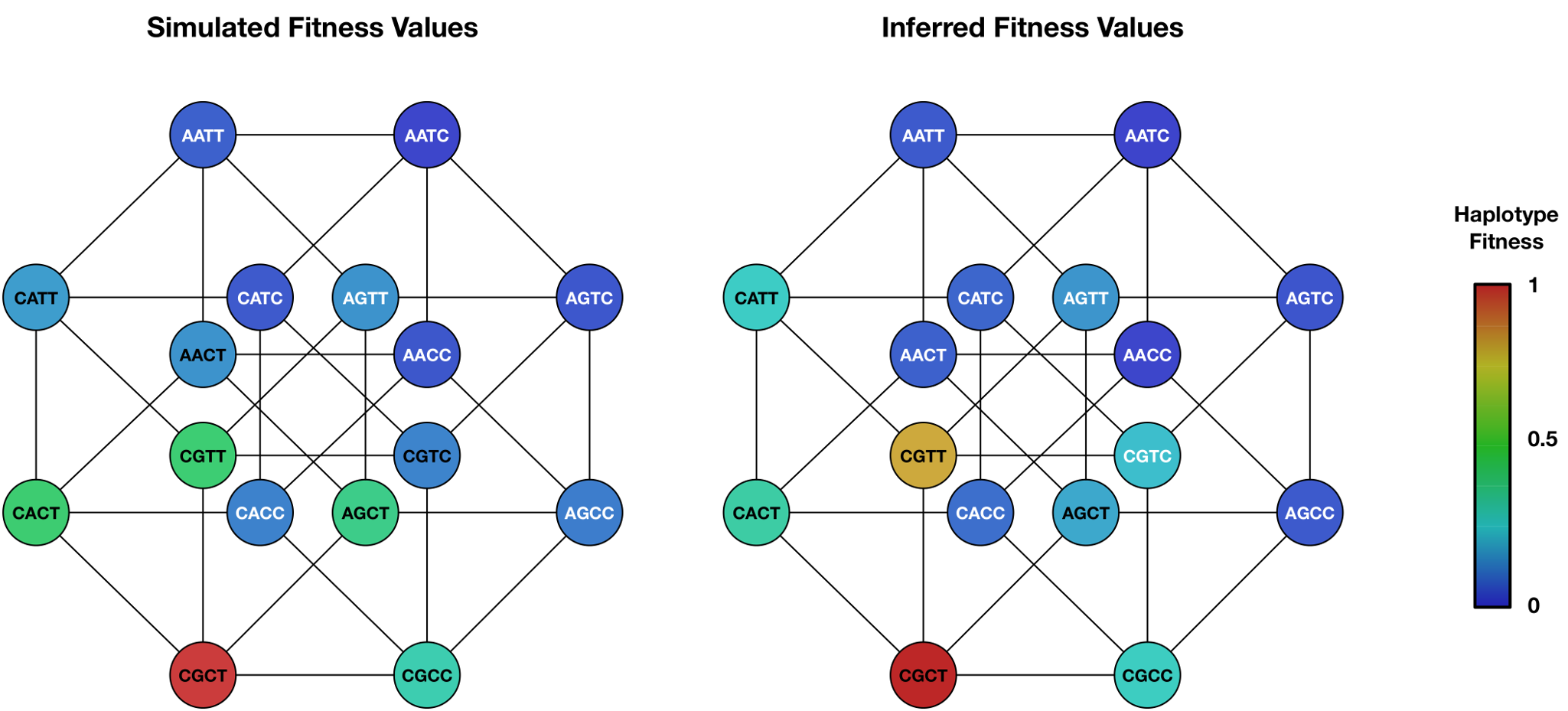

Supplement: S18 Fig — Reported haplotypes show the composition of the viral sequence at the nucleotide positions HA 516, HA 1258, NP 327 and PA 1680 respectively. Colour indicates inferred relative fitness from blue (0) to red (1). Lines indicate haplotypes accessible via a single mutation. (TIF) [file ppat.1006203.s019.tif]

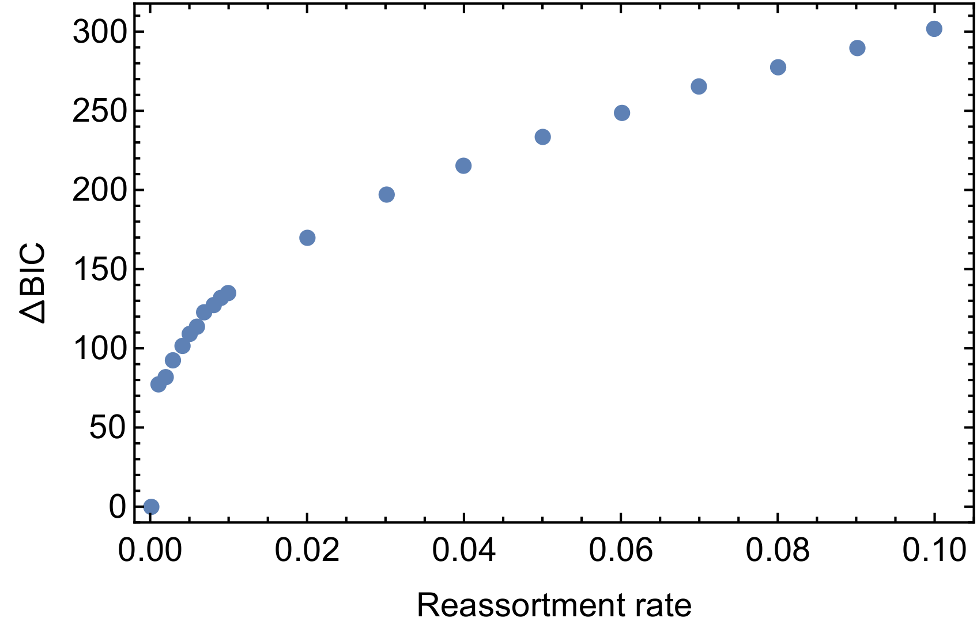

Supplement: S19 Fig — BIC values from the MGML model, relative to the optimal value, for the combined dataset. The data give a close qualitative fit to the values obtained under a multi-parental model. (TIF) [file ppat.1006203.s020.tif]
